# Automated diabetic retinopathy grading and screening using deep learning

**DOI:** 10.1186/s40942-026-00868-5

**Published:** 2026-06-30

**Authors:** Ahmed M. Nashaat Ali Rady, Mohamed A. Azim, Ahmed AbdelMoety, Mohamed Abdelmoaty Ahmed

**Affiliations:** 1 Department of Ophthalmology, Kazan Federal University, Cairo Branch, Cairo, Egypt; 2 Department of Computer Science, College of Computer and Cyber Sciences, University of Prince Mugrin, Madina, Saudi Arabia; 3 Electronics Technology Department, Faculty of Technology and Education, Beni-Suef University, Beni-Suef, 62511 Egypt; 4 Electrical Engineering Department, Faculty of Engineering, South Valley University, Qena, 83523 Egypt; 5 Faculty of Medicine, Merit University, Sohag, Egypt

**Keywords:** Artificial intelligence in ophthalmology, Binary classification, Deep learning, Diabetic retinopathy, Medical image analysis, Multi-class classification

## Abstract

**Purpose:**

To develop and benchmark a unified Deep Learning (DL) pipeline for automated detection and five-level grading of Diabetic Retinopathy (DR), and to derive a high-performance binary screening endpoint (DR vs No DR) suitable for scalable use in resource-limited settings.

**Methods:**

A publicly available five-class DR fundus dataset of 3,500 color photographs graded using the International Clinical Diabetic Retinopathy (ICDR) scale was used. A standardized workflow was applied across eight Convolutional Neural Network (CNN) architectures (AlexNet, Densely Connected Convolutional Network 121 (DenseNet121), Residual Network 50 (ResNet50), eXtreme Inception (Xception), Mobile Network Version 2 (MobileNetV2), Efficient Network Version 2 B2 (EfficientNetV2B2), Inception Version 3 (InceptionV3), Visual Geometry Group 16 (VGG16)), including a 70/20/10 train/validation/test split, optional histogram-based contrast enhancement, strong on-the-fly augmentations, and class-balanced sampling. Seven architectures (DenseNet121, ResNet50, Xception, MobileNetV2, EfficientNetV2B2, InceptionV3, and VGG16) were initialized using ImageNet-pretrained weights and trained using a two-stage transfer-learning strategy with backbone freezing followed by partial fine-tuning, while AlexNet was implemented as a custom architecture and trained from randomly initialized weights.

**Results:**

For five-class grading, VGG16 achieved the highest accuracy (0.7686) and weighted Jaccard index (0.6572), while EfficientNetV2B2 provided the best balanced accuracy (0.6128) and macro Area Under the Receiver Operating Characteristic Curve (AUROC 0.9158). Misclassifications were concentrated between adjacent severity levels. For binary DR screening, modern backbones achieved ≥0.94 accuracy and balanced accuracy; selected models reached AUROC ≈0.982–0.990 and Area Under the Precision–Recall Curve (AUPRC) ≈ 0.987–0.992.

**Conclusion:**

The proposed DL pipeline delivers robust multiclass DR grading and highly discriminative binary screening using widely available CNN backbones. Operating-point calibration enables sensitivity-oriented triage or specificity-oriented confirmation, supporting teleophthalmology and task-shifted DR screening programs to expand coverage and reduce preventable vision loss.

**Clinical trial number:**

Not applicable.

## Introduction

A significant health issue, Diabetic Retinopathy (DR) is a progressive microvascular complication of diabetes, and a significant cause of global visual impairment and blindness, which contributes substantially to the global burden of visual impairment. Its load is especially severe among the working-age populations, where the disease often remains asymptomatic in its early stages and, in case of its unidentified or untreated presence, can lead to irreversible visual impairment.

### Clinical burden of diabetic retinopathy

DR is considered as one of the leading causes of preventable vision loss among working-age adults in the world, where the estimated number of individuals with diabetes retinopathy in 2010 was 126.6 million, and this number is expected to increase to over 191 million in 2030 unless suitable measures are taken to counter the same [1]. DR is not symptomatic at early stages but with progression, irreversible vision loss may occur because this disease is not diagnosed and treated [2]. Timely prevention of vision loss due to DR through systematic screening is very important as it can be avoided in up to 98% of cases [3].

Notwithstanding international screening recommendations, actual screening coverage in low- and middle-income countries, including Egypt, remains variable due to personnel shortages and limitations in healthcare facilities. A population-based study in Sohag Governorate revealed that 85.3% of individuals with diabetes had never received an eye checkup, despite a DR prevalence of 17.9%, including 5.2% with sight-threatening DR and merely 2.3% who had undergone laser therapy [4]. In Qalyubia Governorate, 62.8% of diabetic patients had not undergone a fundus examination, with neglect and lack of awareness recognized as significant obstacles to screening [5]. A retinopathy study conducted in a hospital setting at Cairo University revealed an incidence rate of 20.5% of DR and that majority of the patients were unaware of the visual defects related to diabetes [6]. The results demonstrate that there is a significant gap in screening access and also indicate that scalable and automated screening technologies are urgently required to contribute to better early detection and reduce the burden of vision loss in Egypt.

Mobile imaging and remote access, a combination with Artificial Intelligence (AI), eliminates challenges related to cost, traveling, and specialist availability. Although the international screening guidelines suggest universal screening, there is inconsistent screening coverage in reality, especially in Low- and Middle-Income Countries (LMICs) due to the inadequacy of personnel and lack of infrastructure [7]. India has approximately 74 million people with diabetes, but millions of people have not been screened on DR, and the outcome is high levels of unidentified conditions that endanger vision [7].

Automated DR screening technologies have emerged as a way out in these challenges. The systems use AI and Deep Learning (DL) to analyze retinal images as accurately as when evaluated by trained physicians [8]. EyeArt, a product based on AI, has demonstrated high sensitivity and specificity (>91%), with over 100,000 interactions with patients, enabling quick and reliable detection of referable DR [9].

Automated tools are particularly influential in resource-limited areas. DL algorithms built for low-cost hardware can enhance screening accessibility for remote and underserved groups [10]. Recent studies from India indicate that AI-based solutions such as AI-based DR Screening System (AIDRSS) attained over 92% sensitivity and demonstrated significant utility in rural areas with restricted access to retina specialists [11].

Additionally, new technologies are facilitating the involvement of non-specialists in the process of screening DR using task-shifting and teleophthalmology a strategy that has proven to be effective and can be scaled in LMICs [12]. This type of AI integration with mobile imaging and remote access can reduce cost, travel, and availability of specialists in the area of obstacles [13].

In summary, DR represents an escalating public health menace worldwide. Although early detection via screening is established to mitigate vision loss, inadequate screening persists as a prevalent concern. This necessitates scalable, automated systems that can enhance clinical workflows, minimize diagnostic delays, and expand access to vision-saving procedures.

### Five-level grading importance

**Fig. 1:**
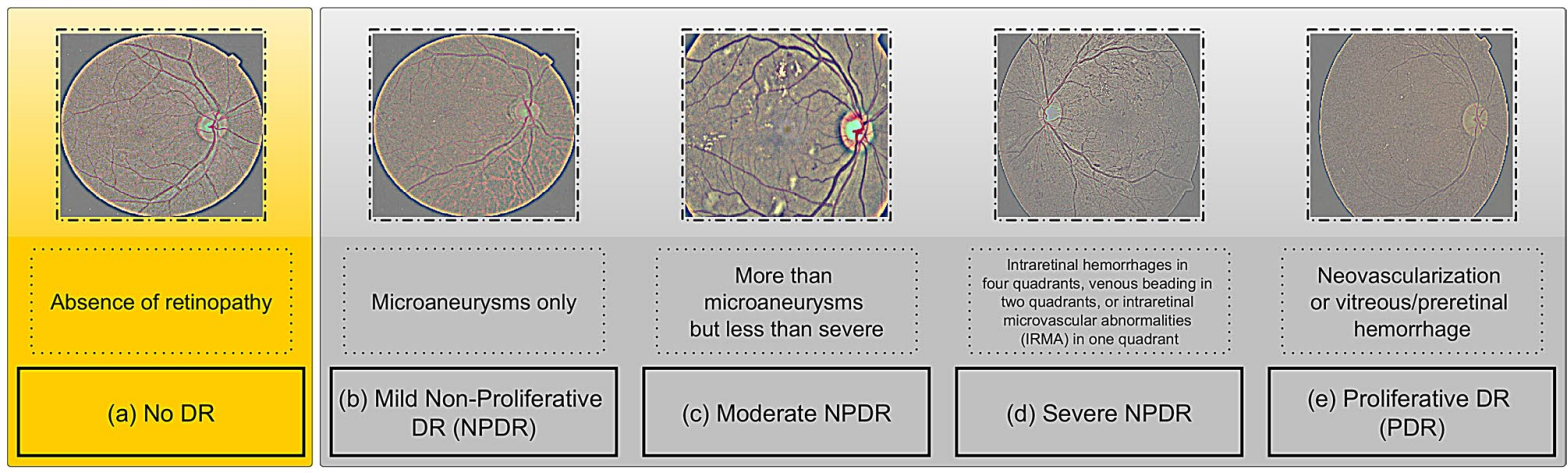
Representative preprocessed images from the five DR classes in the study dataset: (**a**) No DR, (**b**) Mild NPDR, (**c**) Moderate NPDR, (**d**) Severe NPDR, and (**e**) PDR. These images were taken from an APTOS 2019-derived public dataset in which images had already been gaussian filtered and resized to 224 × 224 pixels by the dataset curator before our analysis [17]

DR is a disease of microscopic complication of diabetes that leads to the destruction of the retina under the influence of chronic hyperglycemia, and with different levels of vision loss or even loss, in the absence of diagnosis or treatment [14, 15]. The clinical assessment of DR is regularly founded on grading systems of structured evaluation of the severity of the disease to direct the surveillance, referral and treatment. The five stage severity scale, which was based in the Early Treatment Diabetic Retinopathy Study (ETDRS) and was picked up internationally in the International Clinical Diabetic Retinopathy (ICDR) scale, is the most widely-used [16]. Figure 1 shows representative preprocessed fundus images corresponding to the five DR severity classes: Fig. 1(a) No DR, Fig. 1(b) Mild Non-Proliferative Diabetic Retinopathy (NPDR), Fig. 1(c) Moderate NPDR, Fig. 1(d) Severe NPDR, and Fig. 1(e) Proliferative DR (PDR).

This grading system is not just a descriptive one but with critical implication on clinical management. For example, the decision to refer to a retina specialist is usually made when the NPDR progression turns severe, whereas PDR requires immediate intervention, i.e., panretinal photocoagulation or anti Vascular Endothelial Growth Factor (VEGF) [18, 19]. Both levels also indicate the suggested follow-up period that can be 12 months (mild) or 2–4 months (severe NPDR) based on the risk of progression [19–21].

Moreover, the ordinal relationships are represented by the five-step grading in which each step implies the increase of risk and clinical complexity. This structure is essential in clinical decision and the development of algorithmic models as it enables the system to distinguish between slight variations in severity. The type of Mild NPDR being confused with Moderate NPDR might result in unjustified referrals, whereas the confusion between Severe NPDR and Moderate NPDR may cause delay in saving sight. Recent research confirms that ordinal-aware models significantly outperform flat classifiers by respecting the progression structure of DR, improving both diagnostic accuracy and interpretability [22–24].

### Binary endpoint strategy

Binary DR screening simplifies the problem to a clinically actionable decision: refer (DR present) or reassure (No DR). In population screening programs, this setup is attractive because it maximizes throughput and reduces reading burden—images flagged as DR are escalated for specialist review, while No DR cases are deferred to routine follow-up. In Sub-Section “Five-level grading importance”, the five grades—No DR, Mild NPDR, Moderate NPDR, Severe NPDR, and PDR—collapse naturally into a binary target where “No DR” is 0 and “Mild NPDR, Moderate NPDR, Severe NPDR, PDR” is 1 (DR).

Several recent studies have explored this binary formulation with diverse DL strategies, as summarized in Table 1.

**Table 1:** DL studies focused on binary classification of DR (No DR vs DR)

Reference	Year	Technique Used	Study Description	Major Findings
[25]	2024	Transfer Learning (ResNet101)	Binary classification using 20 pre-trained models; focused on No DR vs DR with image pre-processing and denoising.	ResNet101 achieved 97.33% accuracy, showing high effectiveness across datasets.
[26]	2022	VGG-19 + PSO + SVM	Used VGG-19 with Particle Swarm Optimization and SVM for DR classification on the APTOS 2019 dataset	Achieved 93% accuracy, 99.21% sensitivity, and 98.07% specificity.
[27]	2023	Multi-level Fine-Tuned CNNs (Xception, DenseNet121, MobileNetV2)	Compared fine-tuned models for binary DR classification using pre-trained CNNs.	Xception network reached 97.95% accuracy, outperforming other models.
[28]	2022	Binary CNN	Developed a lightweight CNN for memory-efficient DR vs. non-DR classification	Reduced memory usage and execution time, suitable for edge devices.
[29]	2021	Texture Features + ResNet/DenseNet/DetNet	Combined LBP texture features with DL classifiers for binary DR detection.	ResNet achieved 96.35% accuracy, outperforming other configurations.

A number of recent studies emphasize the usefulness of framing DR screening as a binary outcome (No DR vs DR), particularly in high throughput triage workflows. For example, Saproo et al. found that transfer learning using ResNet101, image pre-processing, and denoising can reach an extremely high accuracy (97.33) when detecting binary DR on datasets [25]. Other researches indicate that hybrid pipelines can also be good performers. On Asia Pacific Tele-Ophthalmology Society (APTOS) 2019 dataset, Lakhera and Garg achieved 93% accuracy with VGG-19 feature extraction and PSO-based selection and an SVM classifier with very high sensitivity (99.21) and specificity (98.07) [26]. On the same note, Beghriche et al. compared various fine-tuned Convolutional Neural Networks (CNNs) backbones and discovered that eXtreme Inception (Xception) was the most accurate with 97.95% accuracy, compared to Densely Connected Convolutional Network 121 (DenseNet121) and Mobile Network Version 2 (MobileNetV2) in their binary setup [27]. In addition to accuracy, efficiency has also been addressed. Kolla and Venugipal suggested a light binary CNN to minimize the memory consumption and execution time to be used on the edge devices [28]. Meanwhile, Adriman et al. established that texture descriptors Local Binary Patterns (LBP) can be used together with deep classifiers to improve discrimination, and a ResNet-based system attains 96.35% accuracy [29].

### Deep learning in fundus imaging

Fundus imaging has long served as a standard, non-invasive tool for diagnosing and monitoring retinal diseases like DR. Over the past few years, a powerful trend has seen DL particularly CNNs dominate as an automated method of detecting, grading, and quantifying DR of fundus images. This paradigm shift has seen a major breakthrough in the speed and cost and scalability of diagnostics, especially in resource-constrained environments.

Retinal fundus DL models have proven to be highly accurate and robust in disease presence and severity detection, in fact, in some instances being more accurate than traditional image processing, and even comparable to human-level performance [30]. With the help of such models, automated screening workflows are possible, which can identify referable DR with high sensitivity and specificity and significantly increase the rate of early diagnosis [31]. Moreover, transfer learning and ensemble modeling are methods of DL that have improved generalizability of models between populations and imaging conditions [32].

### Study objectives, core contributions, and novelty

**Fig. 2:**
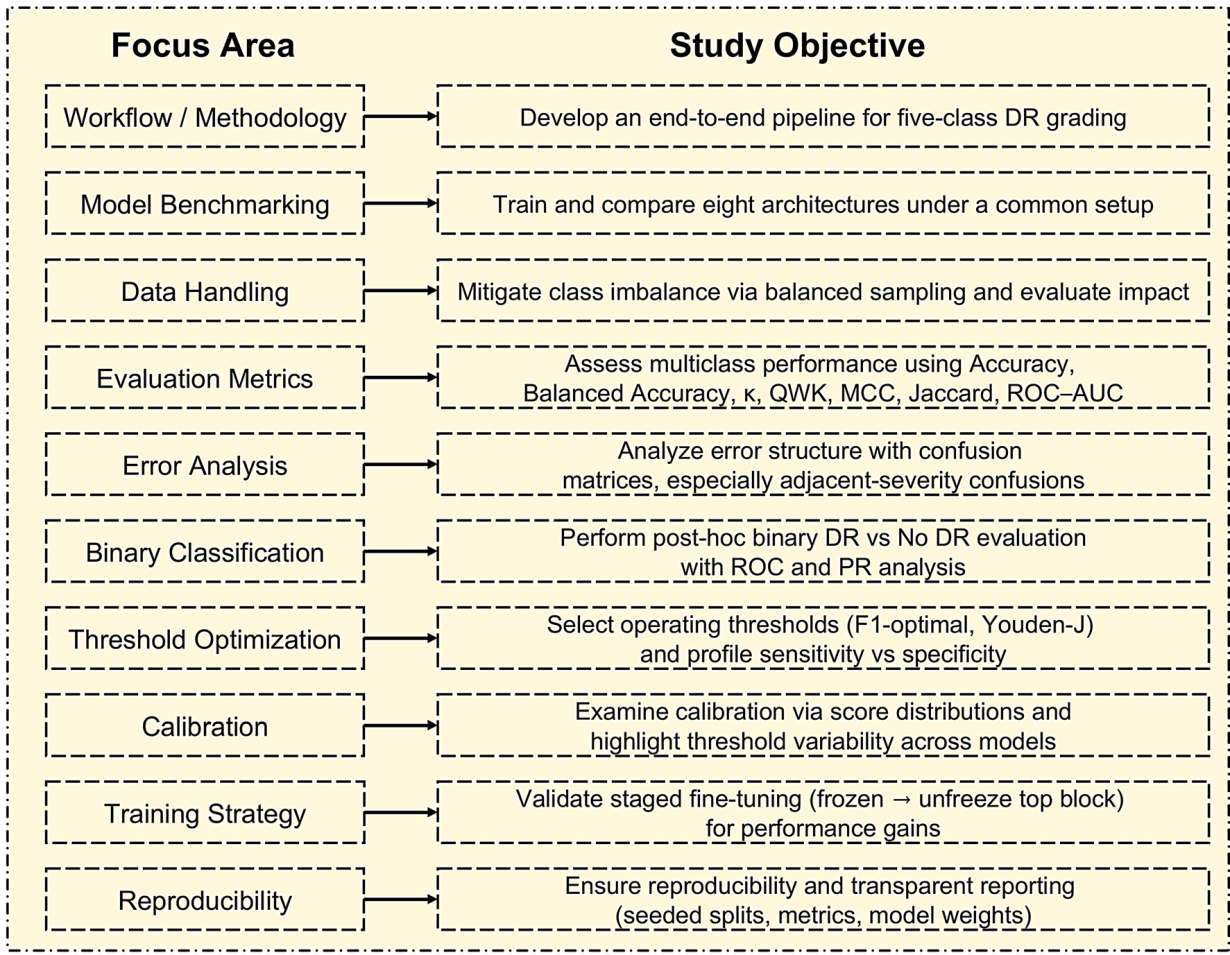
Focus areas and study objectives in DR detection and grading

**Fig. 3:**
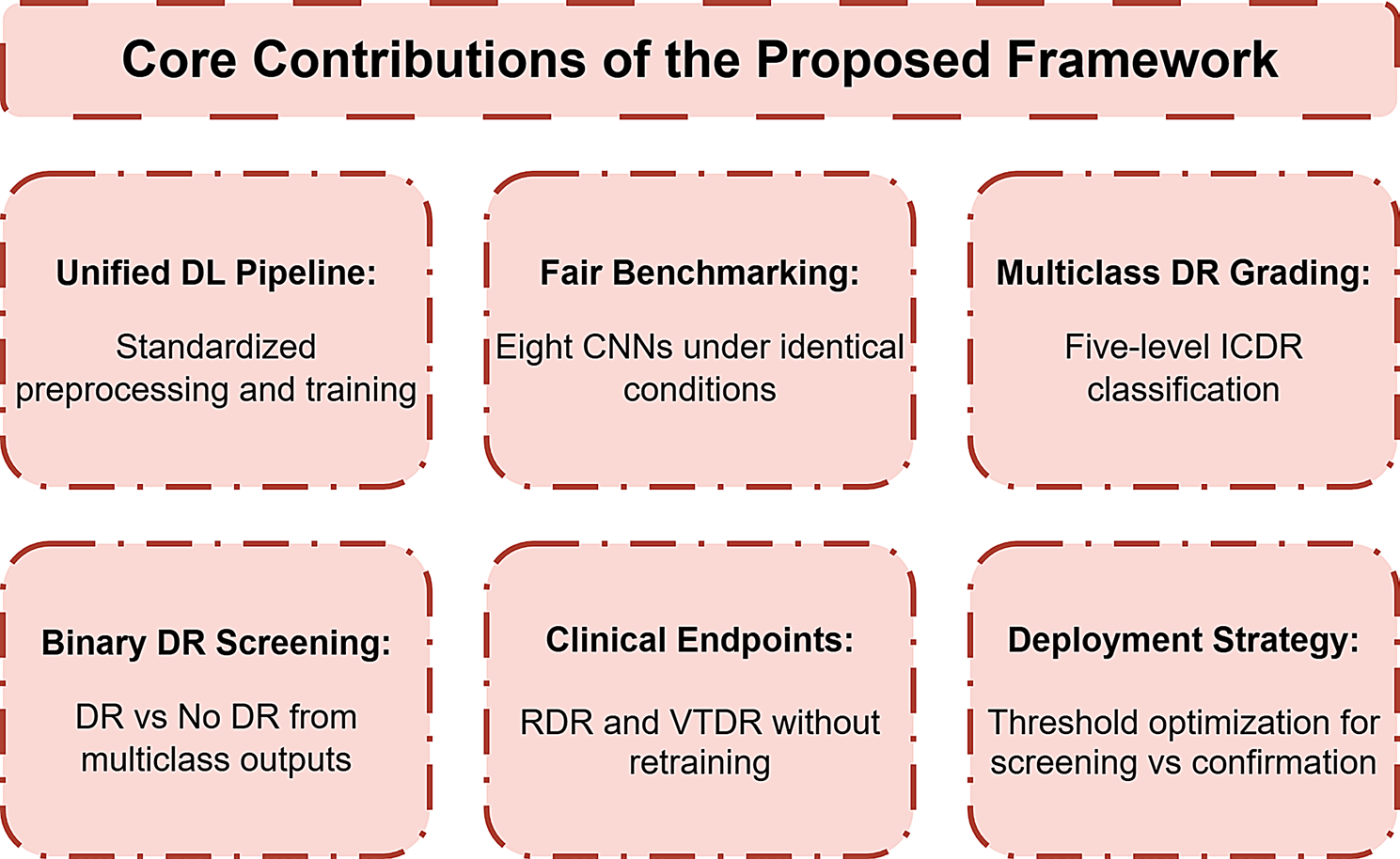
Core contributions of the proposed DR detection and grading framework

The study objectives were systematically organized into major focus areas covering workflow, model benchmarking, data handling, evaluation, and reporting, as summarized in Fig. 2. The core contributions of the proposed framework are further summarized in Fig. 3.

Figure 3 summarizes the core contributions of the proposed framework, including standardized benchmarking, multiclass grading, binary screening, clinically relevant endpoint derivation, and deployment-oriented threshold optimization.

## Methodology

### Dataset description and data split

For this study, the APTOS 2019 Blindness Detection dataset, sourced from Kaggle (Karthik, Maggie, and Sohier Dane, 2019) [33]. It is a dataset of DR that can be identified in retinal images, and with the help of it, early diagnostics and the prevention of blindness are possible because of the 3500 images of DR. The data is freely accessible under the “Creative Commons Zero (CC0): Public Domain” license. In the released version used in this study, the images had already been Gaussian filtered and resized to 224 × 224 pixels by the dataset curator prior to our analysis [17].

The data is classified into five different categories that are determined by the severity of DR, which includes No DR, Mild NPDR, Moderate NPDR, Severe NPDR, and Proliferative DR.

**Fig. 4:**
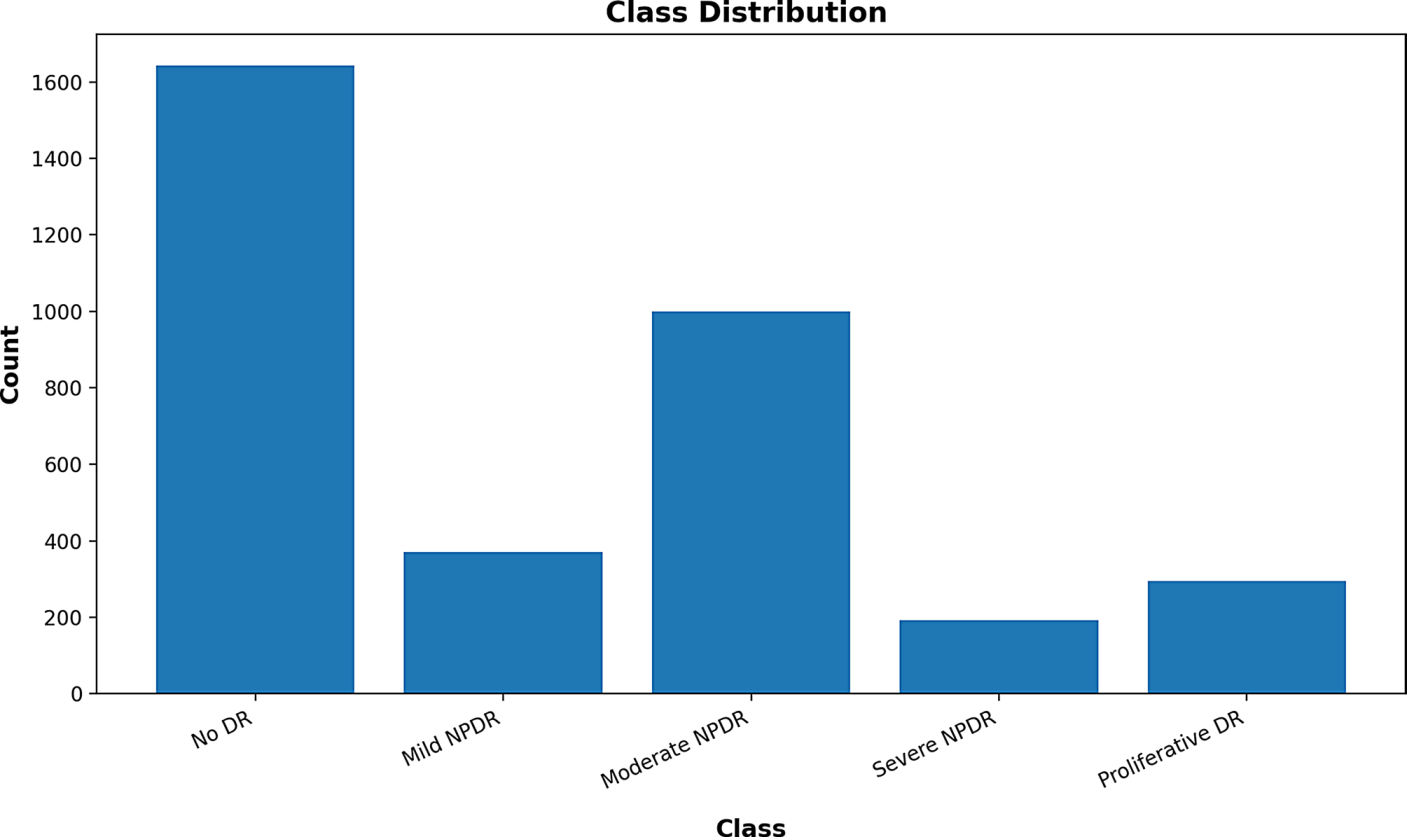
Class distribution of DR dataset

The composition of the dataset in terms of classes is also very skewed as depicted in Fig. 4. The largest category is the No DR class and it consists of 1643 samples (approximately 46.94% of the total dataset) whereas the Severe NPDR and Proliferative DR classes comprise smaller sample sizes, namely 5.51% and 8.43% of the entire data set respectively. Such imbalance in classes also can be a challenge in training a model, where a model will favor the majority class.

The dataset was split into three subsets: Training, Validation, and Test. Table 2 summarizes the distribution of samples across these subsets for each class.

**Table 2:** Dataset split summary across classes (train, validation, test)

Class	Total Samples	Training Set Samples	Validation Set Samples	Test Set Samples	Total Percentage (%)	Training Set Percentage (%)	Validation Set Percentage (%)	Test Set Percentage (%)
No DR	1643	1150	329	164	46.94	46.94	47	46.86
Mild NPDR	370	259	74	37	10.57	10.57	10.57	10.57
Moderate NPDR	999	699	200	100	28.54	28.53	28.57	28.57
Severe NPDR	193	136	38	19	5.51	5.55	5.43	5.43
PDR	295	206	59	30	8.43	8.41	8.43	8.57

Table 2 shows that the Training set contains 70% of the data (approximately 2450 samples), while 20% is allocated to the Validation set (approximately 700 samples) and 10% to the Test set (approximately 350 samples). This data division enables model development on the training set, hyperparameter tuning and model selection on the validation set, and final evaluation on previously unseen test data.

Overall, the sample is made up of 3500 retinal images in five classes of DR severity. Despite an overwhelming imbalance, particularly between the No DR class and the rest, the data has a strong basis to use in training and testing models targeting the detection of DR. This imbalance must be handled with care in order to make sure the model would work at all the severity levels of the condition.

### Overview of the proposed framework

**Fig. 5:**
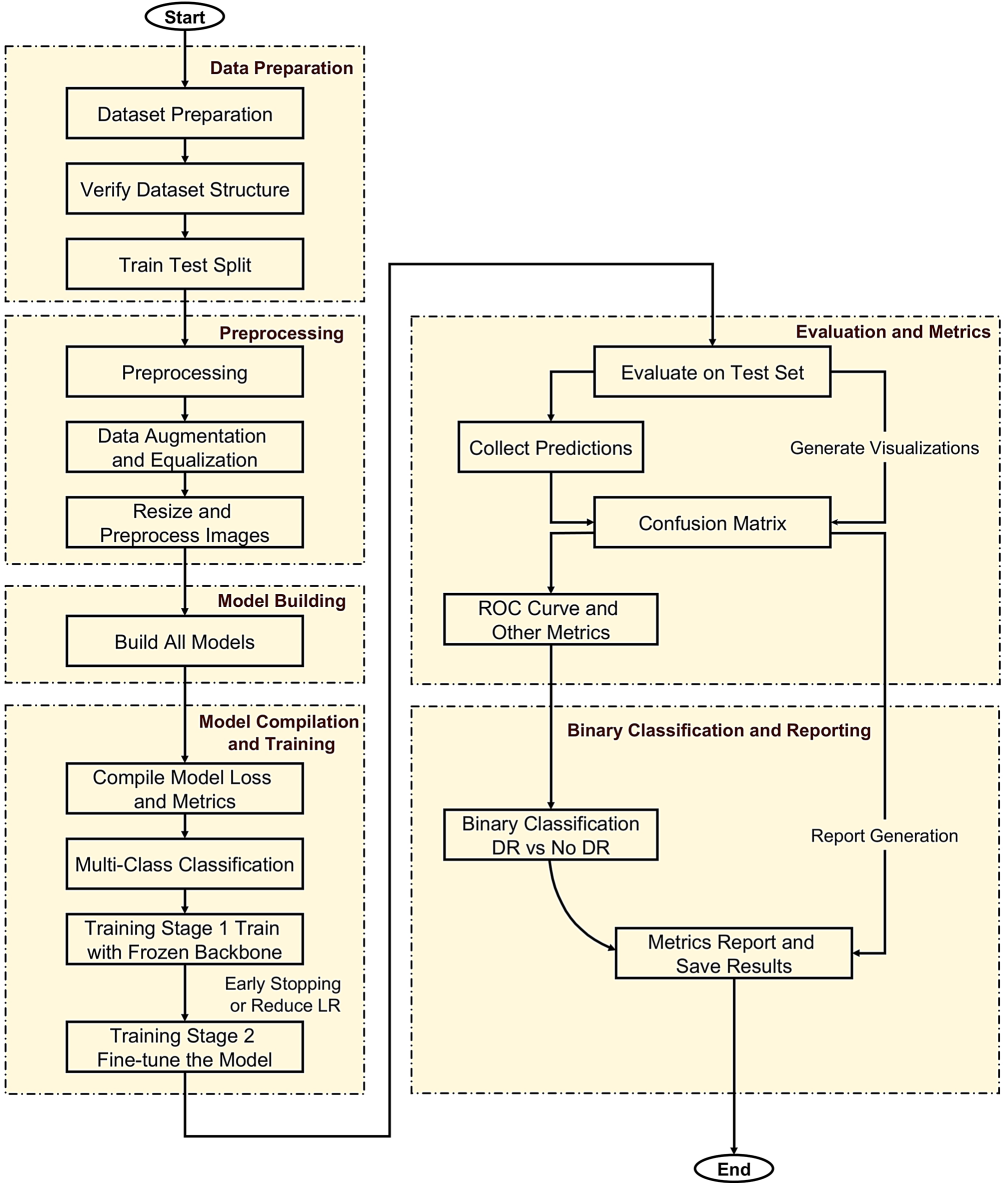
Flowchart for DR detection and grading using DL models

Figure 5 gives the flowchart of the method applied in detecting and grading DR with the help of the DL model. It starts with Dataset Preparation, in which the dataset is validated and divided into training, validation and test sets. Secondly, during the Preprocessing stage, the data is augmented, and histogram-based contrast enhancement is enabled when supported by the preprocessing backend to improve image contrast and input consistency. The next stage is the Model Building phase, and this is followed with the formation of several DL models to classify the DR cases. The next stage after model creation is the Model Compilation and Training stage, where each model was compiled using the specified optimizer, loss function, and metrics before multiclass classification training. The training procedure was performed in two stages for the pretrained models: first, the backbone was frozen and only the classification head was trained; second, the deepest architecture-specific convolutional blocks were unfrozen and fine-tuned using a lower learning rate. In contrast, AlexNet was implemented as a custom architecture and trained from randomly initialized weights. The Evaluation and Metrics stage is performed after the training to measure the effectiveness of the model that involves the generation of predictions, confusion matrices, and Receiver Operating Characteristic (ROC) curves in order to evaluate model accuracy. Lastly, the Binary Classification and Reporting block is used to manage DR vs No DR classification, and the results are saved to produce the final report. This is a systematic process of the assessment and proper grading of DR.

The CNN architectures evaluated in this study were AlexNet, DenseNet121, ResNet50, Xception, MobileNetV2, EfficientNetV2B2, InceptionV3, and Visual Geometry Group 16 (VGG16). DenseNet121, ResNet50, Xception, MobileNetV2, EfficientNetV2B2, InceptionV3, and VGG16 were initialized using ImageNet-pretrained weights and trained within a transfer-learning framework. In contrast, AlexNet was implemented as a custom architecture and trained from randomly initialized weights. For the pretrained models, Stage 1 used a frozen backbone with training limited to the classification head, followed in Stage 2 by partial fine-tuning of the final convolutional block using a lower learning rate.

### Experimental configuration overview

#### General experimental settings

A common set of hyperparameters was established to train and evaluate the models, in order to have comparable results and guarantee consistency of experimentation across the DL architectures. These hyperparameters are related to preparation of the datasets, training schedules, optimization plans, how data is handled and fine-tuning. Tables 3, 4, 5 and 6 present an informative overview of the chosen values and the roles they play in the experiment.

#### Dataset and input parameters

The dataset and input configuration used throughout the experiments are summarized in Table 3.

**Table 3:** Dataset and input parameters for DR classification

Parameter	Value	Description
Image Size	(224, 224)	All images were resized to this dimension.
Data Split	70%/20%/10%	The dataset was split into Train/Validation/Test sets.
Reproducibility Seed	42	Used for random, numpy, and tensorflow to ensure consistent splits and weights.

The input resolution of (224 × 224) was selected to ensure consistency across all evaluated architectures and compatibility with ImageNet-pretrained weights. This resolution provides a practical balance between computational efficiency and diagnostic performance, particularly for scalable deployment scenarios. While higher resolutions may capture finer retinal details, exploring multi-scale or larger input sizes is left for future work.

#### Training schedule parameters

The training schedule was standardized across all evaluated architectures to ensure consistent optimization and fair model comparison, as summarized in Table 4.

**Table 4:** Training schedule parameters

Parameter	Value	Description
Batch Size	32	Number of samples per gradient update.
Stage 1 Epochs	10	Max epochs for initial training. For pretrained models, this stage trained the classification head while the backbone was frozen.
Stage 2 Epochs	100	Max epochs for fine-tuning. For pretrained models, the final convolutional block was partially unfrozen and optimized with a lower learning rate.
Early Stopping Patience	8	Number of epochs with no improvement on val_loss before stopping.
Monitored Metric	val_loss	Metric used for Early Stopping and ReduceLROnPlateau callbacks.

#### Optimizer and loss function settings

The optimizer and loss function settings were defined consistently for the training and fine-tuning stages, as summarized in Table 5.

**Table 5:** Optimizer and loss function settings

Parameter	Value	Description
Loss Function	SparseCategoricalCrossentropy	Used for multi-class classification with integer labels.
Stage 1 Optimizer	Adam	
Stage 1 Learning Rate	1.00E-04	Initial learning rate for the frozen backbone stage.
Stage 2 Optimizer	Adam	
Stage 2 Learning Rate	1.00E-05	Reduced learning rate for the fine-tuning stage.

#### Data handling, augmentation, and fine-tuning parameters

The data handling, augmentation, and fine-tuning settings were configured to improve class balance, input consistency, and model generalization, as summarized in Table 6.

**Table 6:** Data handling, augmentation, and fine-tuning parameters

Parameter	Value	Description
Training Sampler	Balanced Oversampling	(USE_BALANCED_SAMPLING = True) Training samples were drawn from class-specific datasets with approximately equal class-sampling probabilities to reduce class imbalance during training.
Preprocessing	Optional contrast enhancement	Applied when backend was available.
Augmentations	Strong	(STRONG_AUGS = True) Included RandomFlip, RandomRotation, RandomZoom, RandomContrast, RandomTranslation, and RandomBrightness.
Unfreeze Scope	Deepest Block Only	(UNFREEZE_CONV4 = False) For Stage 2, only the final convolutional block (e.g., block5_ for Visual Geometry Group 16 (VGG16), conv5_ for Residual Network 50 (ResNet50)) was unfrozen.
Backbone Initialization	Mixed	DenseNet121, ResNet50, Xception, MobileNetV2, EfficientNetV2B2, InceptionV3, and VGG16 were initialized with ImageNet-pretrained weights, whereas AlexNet was trained from random initialization.

### Evaluation metrics

In order to measure the performance of the DL models, a number of metrics were employed to measure the multiclass and binary classification tasks, with emphasis placed on the separation of the models on discriminating that of “DR” and that of “No DR”.

#### Multiclass classification metrics

**Accuracy** quantifies overall correctness as the proportion of correctly classified samples out of the total, as defined in Eq. (1):

1 $$Accuracy = {{True\,Positives + True\,Negatives} \over {Total\,Samples}}$$

Because class prevalence can be imbalanced, **Balanced Accuracy** averages class-wise sensitivity and specificity, as defined in Eq. (2):

2 $$\begin{gathered} Balanced\, Accuracy = \frac{1}{2}\left( {\frac{{True\,Positives}}{{True\, Positives + False\, Negatives}}} \right) \hfill \\ \quad \quad \quad \quad \quad \quad \quad \quad \quad + \left( {\frac{{True\, Negatives}}{{True\, Negatives + False\, Positives}}} \right) \hfill \\ \end{gathered}$$

To account for agreement beyond chance, **Cohen’s Kappa** compares observed accuracy with chance agreement, as defined in Eq. (3):

3 $$Cohen's\,Kappa = {{{P_o} - {P_e}} \over {1 - {P_e}}}$$

Where ${P_o}$ is the observed accuracy, and ${P_e}$ is the expected accuracy by chance.

Because DR severity is ordinal, this study also reports QWK, which penalizes larger ordinal misclassifications more heavily, extending the logic of Cohen’s Kappa as defined in Eq. (3).

#### Binary classification metrics (DR vs no DR)

In the binary classification task, it was aimed at differentiating between “DR” and “No DR”. The metrics were the following:

Area Under the Receiver Operating Characteristic Curve (AUROC) summarizes the ROC curve by integrating True Positive Rate (TPR) with respect to False Positive Rate (FPR), as defined in Eq. (4):

4 $$AUC = \mathop \smallint \limits_0^1 TPR\left( x \right)d\left( {FPR\left( x \right)} \right)$$

Where $TPR$ is the true positive rate and $FPR$ is the false positive rate.

Precision measures the proportion of predicted positives that are correct, as shown in Eq. (5), while Recall (sensitivity) measures the proportion of actual positives correctly identified, as shown in Eq. (6):

5 $$Precision = {{True\,Positives} \over {True\,Positives + False\,Positives}}$$

6 $$Recall = {{True\,Positives} \over {True\,Positives + False\,Negatives}}$$

The **F1-Score** is the harmonic mean of Precision and Recall, balancing both measures, as shown in Eq. (7):

7 $$F1-Score = 2 \times {{Precision \times Recall} \over {Precision + Recall}}$$

The Matthews Correlation Coefficient (MCC) incorporates all four categories of the confusion matrix ($TP$, $TN$, $FP$, and $FN$), providing a balanced measure even under class imbalance, as shown in Eq. (8):

8 $$MCC = {{TP \times TN - FP \times FN} \over {\sqrt {\left( {TP + FP} \right)\left( {TP + FN} \right)\left( {TN + FP} \right)\left( {TN + FN} \right)} }}$$

Where $TP$, $TN$, $FP$, and $FN$ represent True Positives, True Negatives, False Positives, and False Negatives, respectively.

Confusion matrices are also provided to visualize the performance of the classifiers, their values can form the foundation of Precision, Recall, F1, and MCC as in Eqs. (5), (6), (7) and (8).

#### Post-hoc analysis for DR vs no DR

The post-hoc binary classification analysis was conducted to evaluate each model’s ability to distinguish between “DR” and “No DR”. Continuous prediction scores $P\left( {DR\mid image} \right)$ were obtained by aggregating all non–No DR classes into a single positive class. Decision thresholds were swept over the predicted test-set probabilities in a post-hoc exploratory analysis, and the operating threshold ${t_{opt}}$ was selected by maximizing the F1-score for the DR-positive class, as defined in Eq. (9). Therefore, the threshold-dependent binary metrics should be interpreted as operating-point analysis rather than as validation-calibrated independent test performance.

9 $${t_{opt}} = \mathop {{\rm{argmax}}}\limits_t F1\left( t \right)$$

This criterion emphasizes a balance between precision and recall for the DR class. For completeness, Youden’s J statistic $\left( {TPR - FPR} \right)$ was also computed as a secondary reference but was not used for threshold selection.

## Results and discussion

This section summarizes empirical findings across four parts. First, this study describes training dynamics and generalization across all eight architectures and shows the convergence behavior, stability, and influence of augmentations, rebalancing, and staged fine-tuning. Second, this study presents the results of multiclass grading performance according to the five levels of DR in terms of accuracy, agreement, ranking and overlap metrics, as well as connects the results with the general error distributions across the successive stages. Third, this study evaluates the derived binary endpoint (DR vs No DR) using F1 maximization to select operating thresholds and compares the accuracy, sensitivity/specificity, predictive values, and discrimination capacity, with attention to calibration and threshold variability across models. Lastly, this paper contrasts architectures more broadly, with a discussion of the accuracy-robustness trade-offs, strength by weaknesses of deeper backbones, and implications in practice of screening versus confirmatory workflows.

### Training performance

**Fig. 6:**
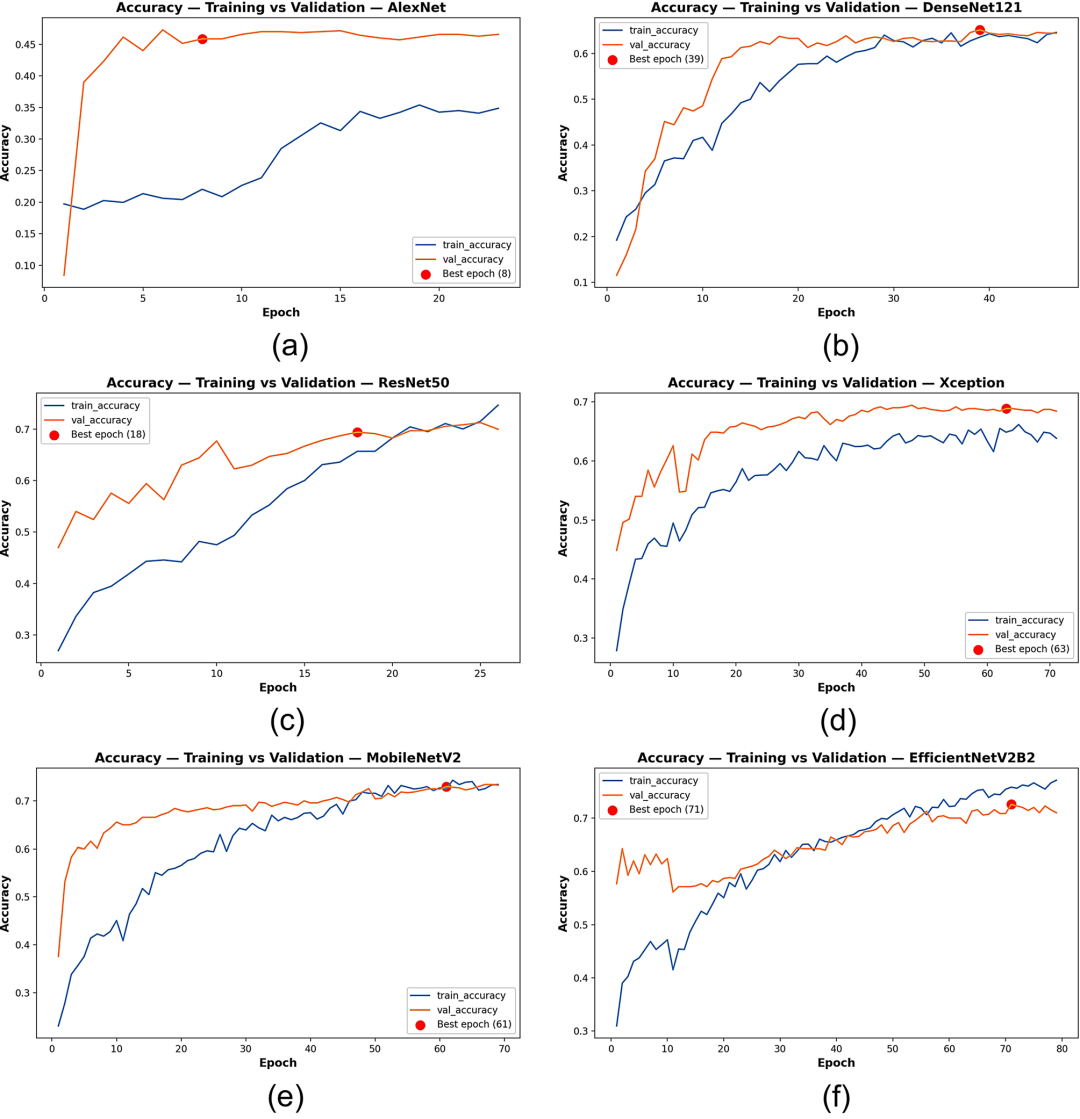

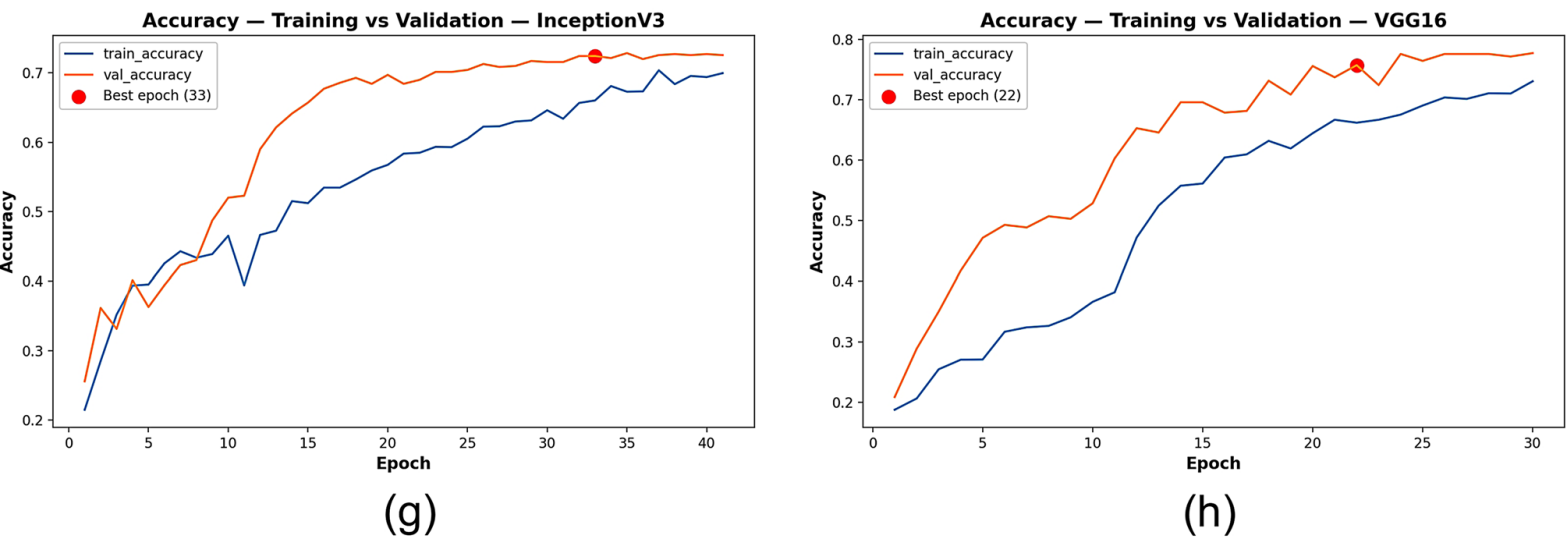
Training vs. validation accuracy for eight DL architectures in DR classification: (**a**) AlexNet; (**b**) DenseNet121; (**c**) ResNet50; (**d**) Xception; (**e**) MobileNetV2; (**f**) EfficientNetV2B2; (**g**) InceptionV3; (**h**) VGG16

**Fig. 7:**
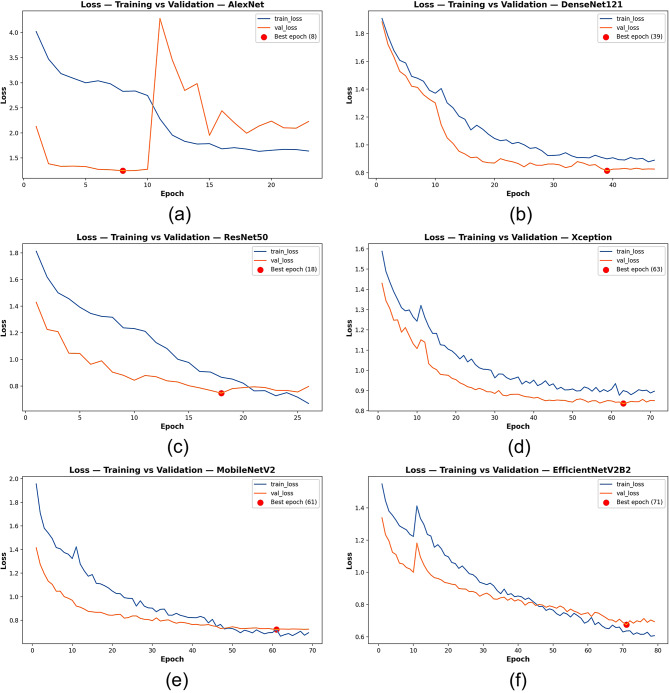

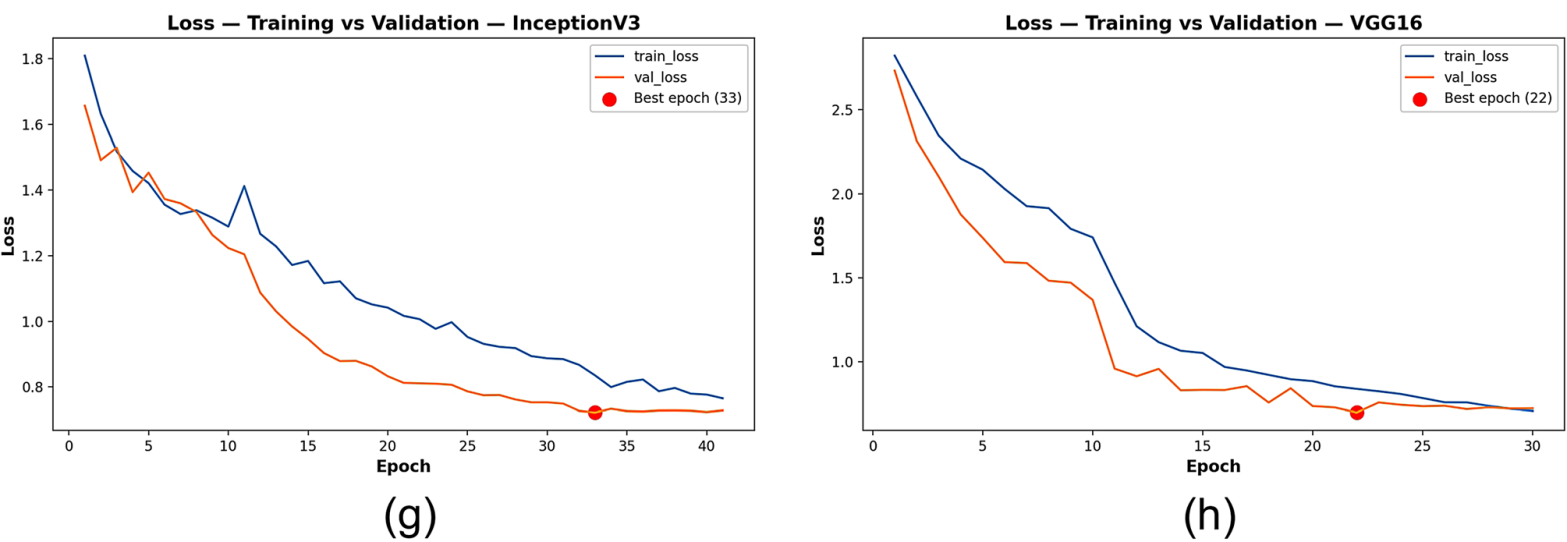
Training vs. validation loss for eight DL architectures in DR classification: (**a**) AlexNet; (**b**) DenseNet121; (**c**) ResNet50; (**d**) Xception; (**e**) MobileNetV2; (**f**) EfficientNetV2B2; (**g**) InceptionV3; (**h**) VGG16

The accuracy curves across the eight architectures show the anticipated trend of rapid early improvement followed by slower gains or stabilization, as shown in Fig. 6. Figure 7 depicts that the associated losses decrease sharply initially and then become flat as optimization is carried out. In Figs. 6(a)–(h) and 7(a)–(h), red markers indicate the best validation checkpoints selected for final evaluation.

The shallow baseline shows clear capacity limits: training and validation accuracies rise only modestly before settling well below the stronger backbones, as shown in Fig. 6(a), while the validation loss stabilizes comparatively high and remains somewhat erratic, as shown in Fig. 7(a). In contrast, DenseNet121 converges quickly to a stable validation plateau with a small train–validation gap, as shown in Fig. 6(b), and maintains a smooth, persistently low validation loss, as shown in Fig. 7(b). ResNet50 improves steadily with a narrow gap between curves, as shown in Fig. 6(c), and its loss traces descend together and level off without late divergence, as shown in Fig. 7(c).

Architectures leveraging depthwise separable or factorized convolutions generalize particularly well. Xception often shows validation accuracy tracking or slightly surpassing training accuracy—an effect expected when strong augmentations make the training task harder—as shown in Fig. 6(d), with validation loss remaining below the training loss for much of training, as shown in Fig. 7(d). MobileNetV2, despite its lightweight design, climbs gradually to a competitive and stable plateau, as shown in Fig. 6(e), while its loss continues to drift downward late in training, as shown in Fig. 7(e). Efficient Network Version 2 B2 (EfficientNetV2B2) follows a slow-and-steady trajectory with accuracy improving across many epochs before peaking, as shown in Fig. 6(f), and validation loss decreasing deep into training, as shown in Fig. 7(f). Inception Version 3 (InceptionV3) rises quickly to a high plateau that then stabilizes, as shown in Fig. 6(g), with decisive loss reductions that flatten without notable train–validation separation, as shown in Fig. 7(g).

As the upper blocks are fine-tuned in stages, VGG16 gradually gains and has a distinct best-epoch emerging following unfreezing, as indicated by Fig. 6(h), and the loss decreases and has only slight oscillations at the end as indicated by Fig. 7(h).

Taken together, most models display validation accuracy closely tracking training accuracy and validation loss at or below training loss—consistent with strong augmentation, class rebalancing, and early stopping—as shown in Figs. 6 and 7. Periods when validation accuracy is less than training accuracy such as Xception and at one point DenseNet121 are consistent with the application of on-the-fly augmentations that make training more challenging as indicated in Fig. 6(b) and (d). The initial plateau of shallow baseline as compared to the sustained progress of modern backbones has highlighted the worth of deeper and more regularized feature extractors in the grading of DR as indicated in Figs. 6(a)–(h) and 7(a)–(h).

### Multiclass classification results

The grading task of five stages reveals that there is a significant performance difference between the shallow baseline and the modern backbones. Overall accuracy rises from 0.4600 for AlexNet to 0.7686 for VGG16, with InceptionV3 and EfficientNetV2B2 close behind at 0.7457 and 0.7286, while DenseNet121, ResNet50, Xception, and MobileNetV2 achieve 0.6600, 0.6886, 0.7143, and 0.7257. Balanced accuracy emphasizes cross-class robustness: EfficientNetV2B2 leads at 0.6128, followed by VGG16 at 0.5853, DenseNet121 at 0.5782, ResNet50 at 0.5761, Xception at 0.5686, InceptionV3 at 0.5566, and MobileNetV2 at 0.4521, with AlexNet trailing at 0.2096. Table 7 shows these summary statistics.

**Table 7:** Overall classification performance based on accuracy metrics

Algorithm	Accuracy	Balanced Accuracy
AlexNet	0.4600	0.2096
DenseNet121	0.6600	0.5782
ResNet50	0.6886	0.5761
Xception	0.7143	0.5686
MobileNetV2	0.7257	0.4521
EfficientNetV2B2	0.7286	**0.6128**
InceptionV3	0.7457	0.5566
VGG16	**0.7686**	0.5853

These trends are supported by agreement-based analyses. VGG16 has the highest Cohen’s κ 0.6562 and the largest MCC of 0.6607, whereas InceptionV3 has Cohen’s κ 0.6201 and MCC 0.6228 and EfficientNetV2B2 has Cohen’s κ 0.6082 and MCC 0.6188. Among the remaining modern backbones, Xception (κ 0.5871; MCC 0.5924), MobileNetV2 (κ 0.5784; MCC 0.5863), ResNet50 (κ 0.5556; MCC 0.5632), and DenseNet121 (κ 0.5287; MCC 0.5512) cluster tightly, whereas AlexNet records κ of 0.0199 and MCC of 0.0356. When ordinal severity is emphasized, EfficientNetV2B2 achieves the best quadratic-weighted κ of 0.8294, followed by MobileNetV2 at 0.8017, DenseNet121 at 0.7994, InceptionV3 at 0.7991, VGG16 at 0.7932, Xception at 0.7853, and ResNet50 at 0.7740, far ahead of AlexNet at 0.0382. These agreement statistics are reported in Table 8.

**Table 8:** Agreement-based evaluation of multiclass classification models

Algorithm	Cohen’s κ	Quadratic-weighted κ	MCC
AlexNet	0.0199	0.0382	0.0356
DenseNet121	0.5287	0.7994	0.5512
ResNet50	0.5556	0.7740	0.5632
Xception	0.5871	0.7853	0.5924
MobileNetV2	0.5784	0.8017	0.5863
EfficientNetV2B2	0.6082	**0.8294**	0.6188
InceptionV3	0.6201	0.7991	0.6228
VGG16	**0.6562**	0.7932	**0.6607**

Methods are further differentiated by ranking metrics and overlap. The lowest number of multi-class confusions was seen on the class-balanced and sample-weighted scales, with VGG16 producing the best Jaccard indices (0.4515 macro and 0.6572 weighted). Xception and ResNet50 achieve weighted Jaccard of 0.6023 and 0.5808, respectively, while InceptionV3 and EfficientNetV2B2 follow with weighted Jaccard of 0.6280 and 0.6217, respectively, and macro Jaccard of 0.4342 and 0.4307. DenseNet121 and MobileNetV2 get 0.3752 and 0.3532 for macro with 0.5605 and 0.5952 for weighted, and AlexNet stays at 0.1094 for macro and 0.2329 for weighted. In terms of ranking ability, VGG16 achieved the highest micro-average Area Under the Curve (AUC) of 0.9486, followed by InceptionV3 (0.9447) and EfficientNetV2B2 (0.9408); MobileNetV2, ResNet50, Xception and DenseNet121 appeared to be competitive in the range of 0.9391 to 0.9243; and the lowest was AlexNet with 0.8074. The macro-average AUC reaches a maximum of 0.9158 with EfficientNetV2B2, followed by VGG16 at 0.9149 and DenseNet121 at 0.9146, and finally InceptionV3 at 0.9026, ResNet50 at 0.9003, Xception at 0.8993, MobileNetV2 at 0.8975 and AlexNet at 0.7500. These measurements are presented in Table 9.

**Table 9:** Evaluation of overlap indices and Area Under the ROC Curve (AUC) for multiclass discrimination performance

Algorithm	Jaccard index (macro)	Jaccard index (weighted)	AUC (micro-average)	AUC (macro-average)
AlexNet	0.1094	0.2329	0.8074	0.7500
DenseNet121	0.3752	0.5605	0.9243	0.9146
ResNet50	0.4004	0.5808	0.9308	0.9003
Xception	0.4059	0.6023	0.9279	0.8993
MobileNetV2	0.3532	0.5952	0.9391	0.8975
EfficientNetV2B2	0.4307	0.6217	0.9408	**0.9158**
InceptionV3	0.4342	0.6280	0.9447	0.9026
VGG16	**0.4515**	**0.6572**	**0.9486**	0.9149

**Fig. 8:**
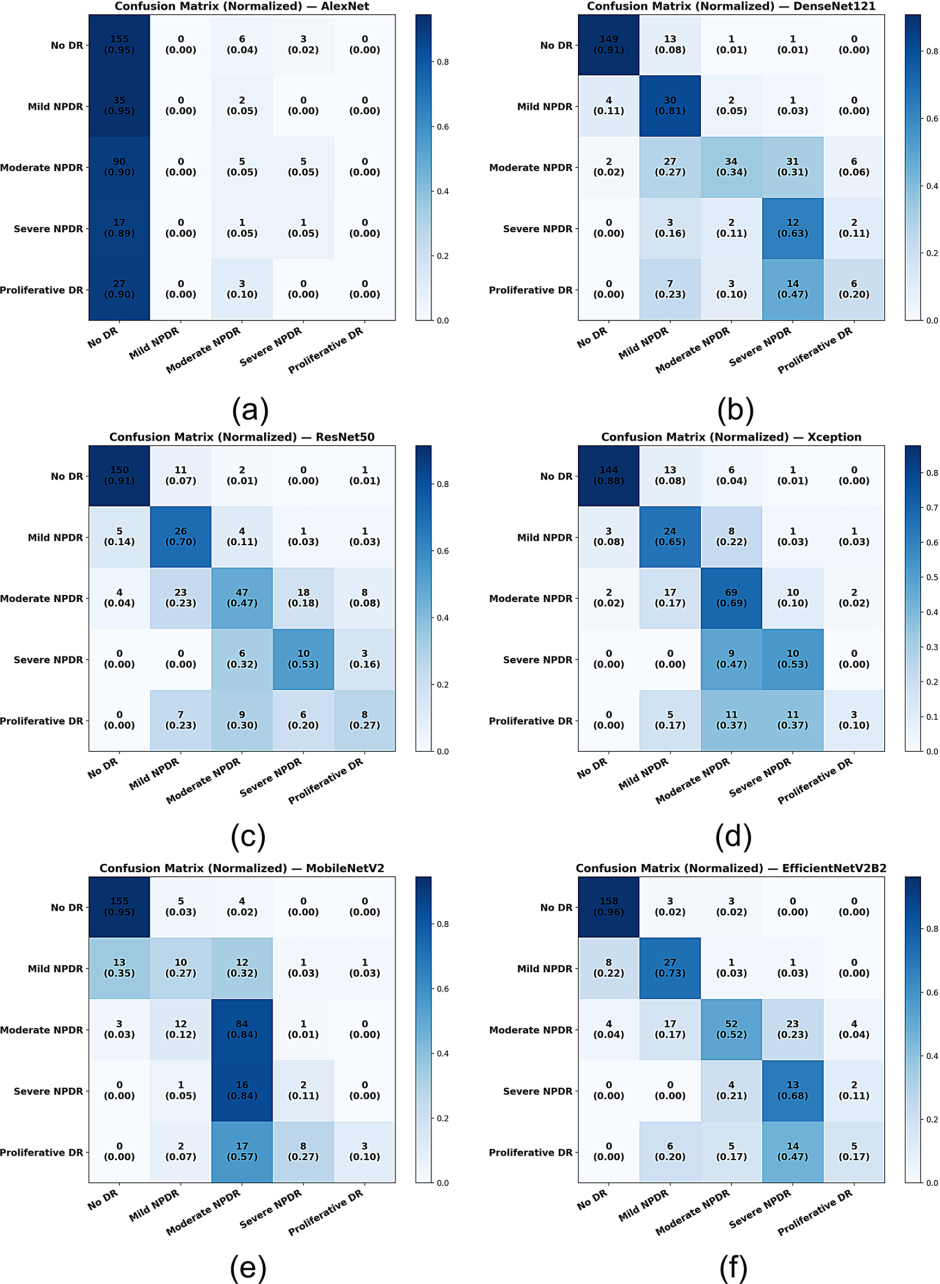

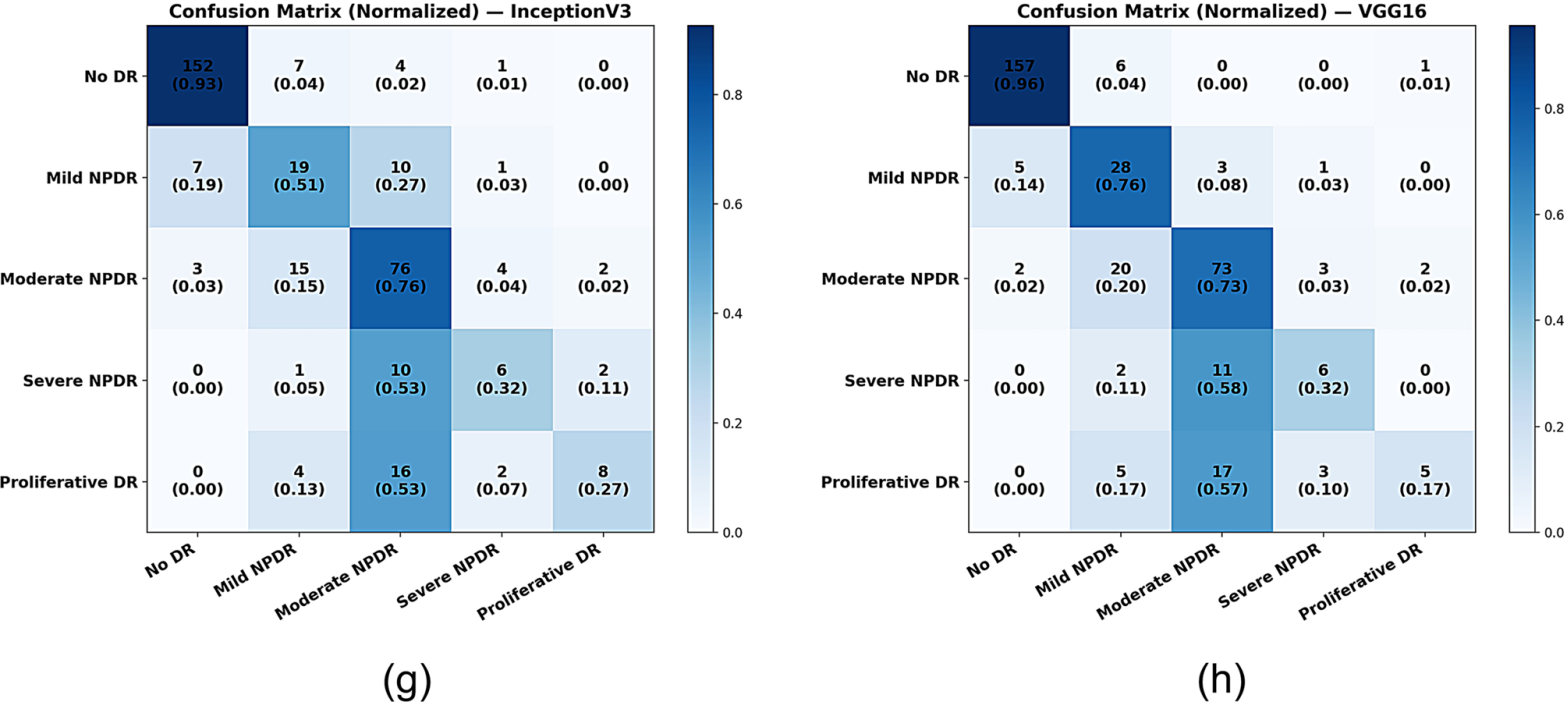
Normalized confusion matrices for five-stage DR grading (No DR, Mild NPDR, Moderate NPDR, Severe NPDR, PDR) using DL architectures: (**a**) AlexNet; (**b**) DenseNet121; (**c**) ResNet50; (**d**) Xception; (**e**) MobileNetV2; (**f**) EfficientNetV2B2; (**g**) InceptionV3; (**h**) VGG16

**Fig. 9:**
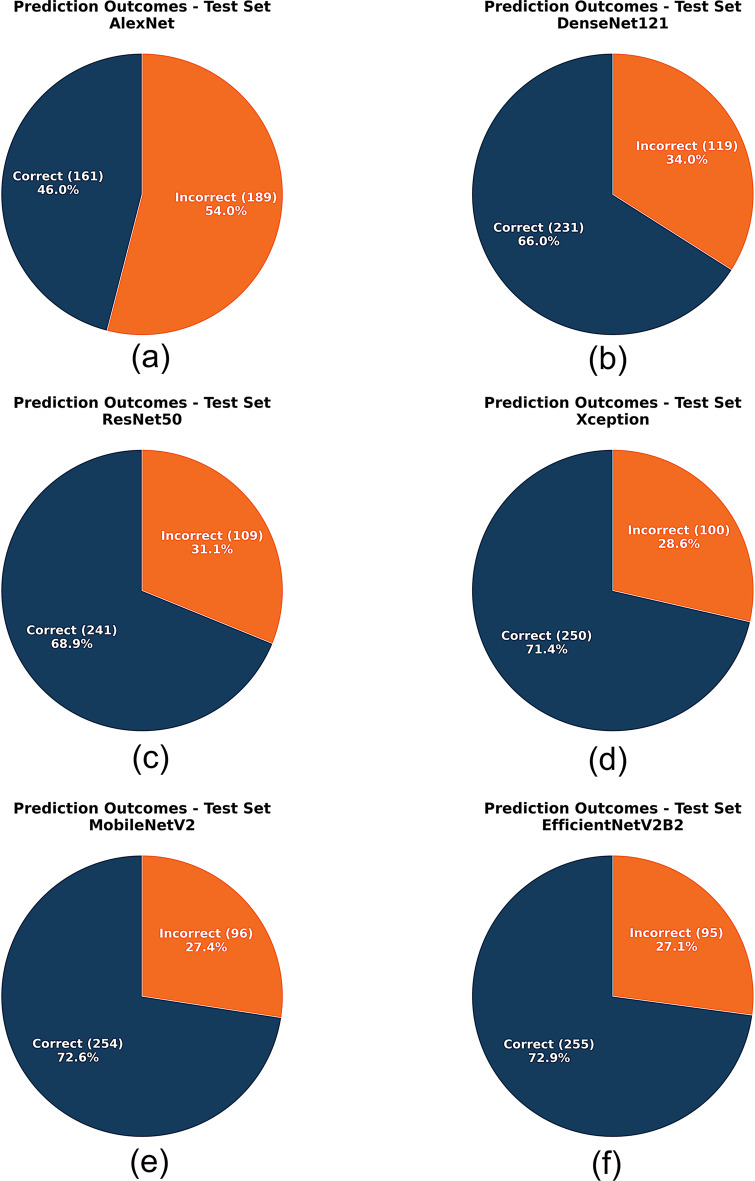

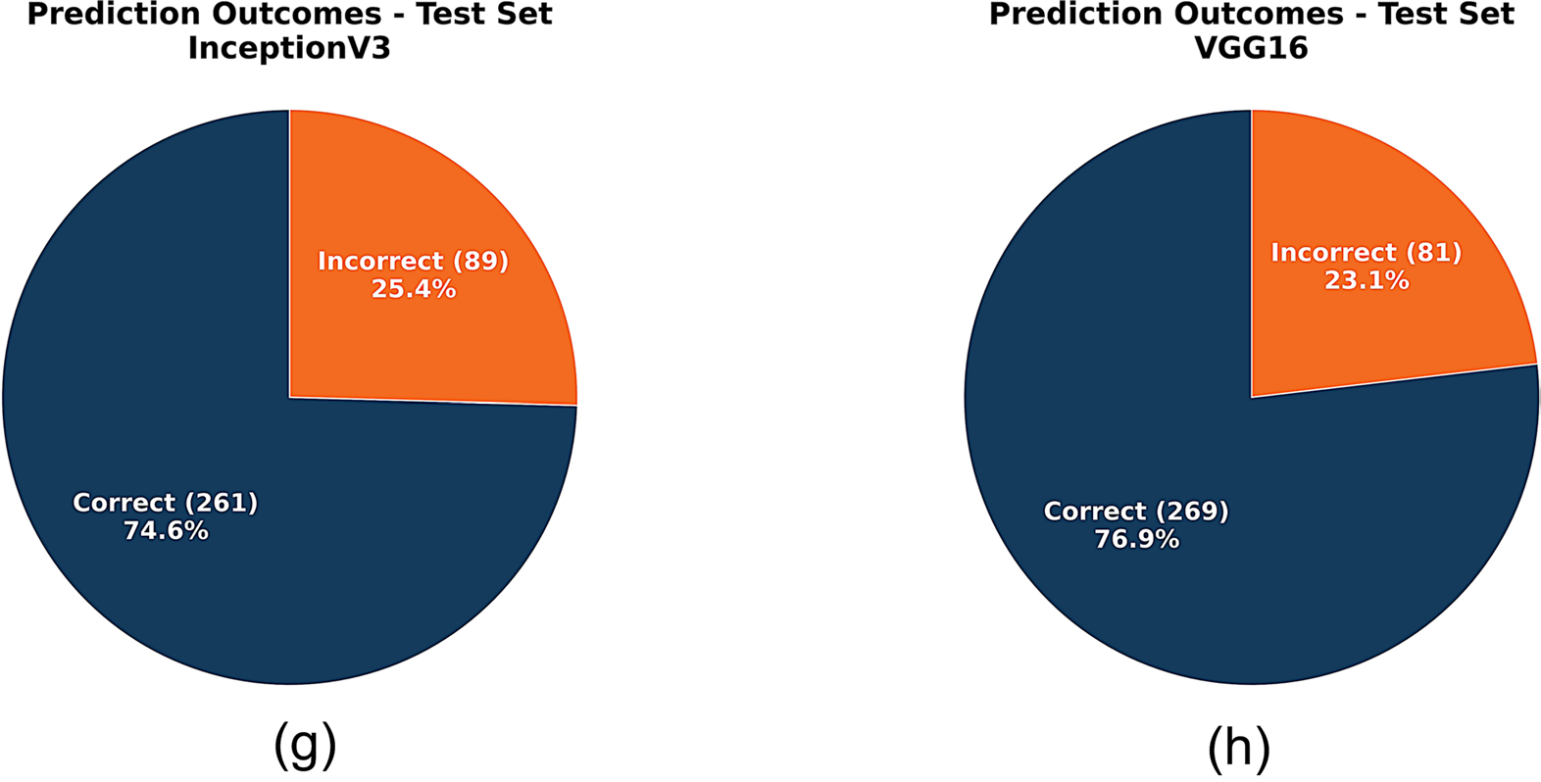
Test-set prediction outcomes (correct vs. incorrect) for five-stage DR grading using DL architectures: (**a**) AlexNet; (**b**) DenseNet121; (**c**) ResNet50; (**d**) Xception; (**e**) MobileNetV2; (**f**) EfficientNetV2B2; (**g**) InceptionV3; (**h**) VGG16

**Fig. 10:**
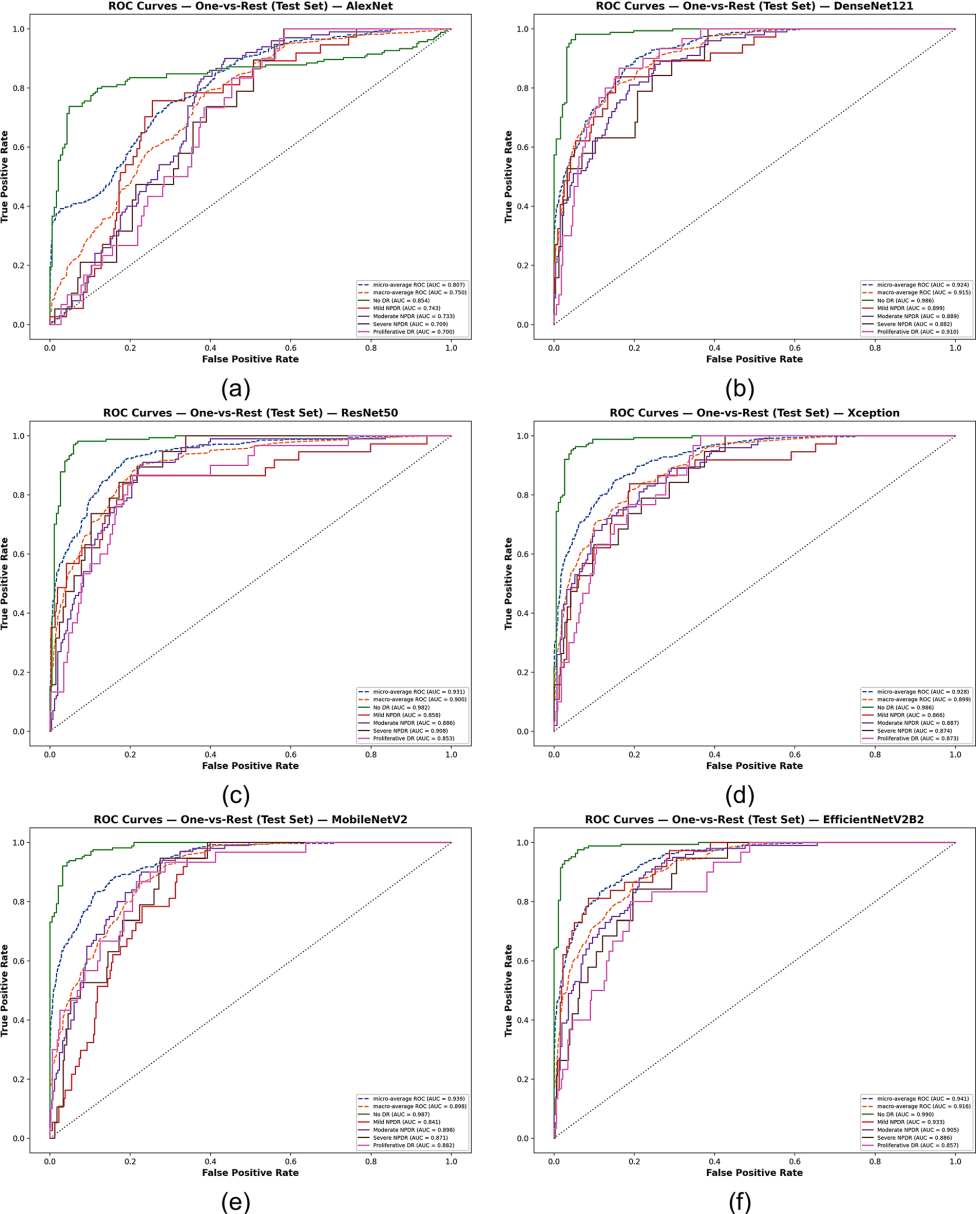

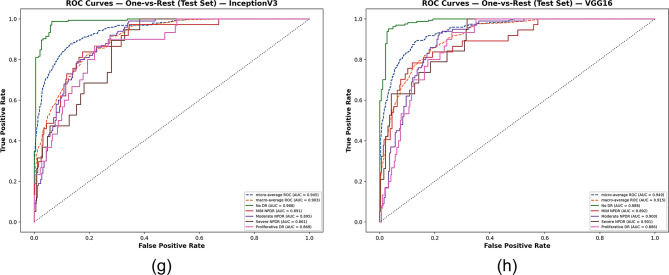
One-vs-rest ROC curves on the test set (micro- and macro-averages included) for five-stage DR grading using DL architectures: (**a**) AlexNet; (**b**) DenseNet121; (**c**) ResNet50; (**d**) Xception; (**e**) MobileNetV2; (**f**) EfficientNetV2B2; (**g**) InceptionV3; (**h**) VGG16

The summaries align with the class-wise error structure. While Fig. 8(a)–(h) shows that No DR is mostly well isolated from other severities by the stronger backbones, most mistakes happen just in adjacent severities—Mild vs. Moderate and Severe vs. Proliferative. Figure 9(a)–(h) show that the percentage of correct predictions steadily rise from AlexNet to the best models: the longest correct segment is for VGG16, whereas InceptionV3 and EfficientNetV2B2 also achieve high proportions of correct prediction. Figure 10(a)–(h) illustrate receiver-operating characteristics showing a strong one-vs-rest separation, with higher micro- and macro-averages for the top architectures and a visibly tighter curves vs shallow baseline.

To summarize, VGG16 provides the highest overall correctness - accuracy 0.7686, micro-AUC 0.9486, and the best inter-class Jaccard indices, whilst EfficientNetV2B2 delivers optimal per-class behavior adhering to the best balanced accuracy 0.6128 and macro-AUC 0.9158. InceptionV3 constantly demonstrates competitiveness across all criteria. The complementary perspectives on accuracy, agreement, overlap, and ROC behavior converge to the conclusion that contemporary deep architectures significantly enhance five-class DR grading, with persistent challenges primarily at the boundaries between adjacent severity levels, as illustrated in Figs. 8, 9, and 10.

### Binary classification results (DR vs no DR)

**Fig. 11:**
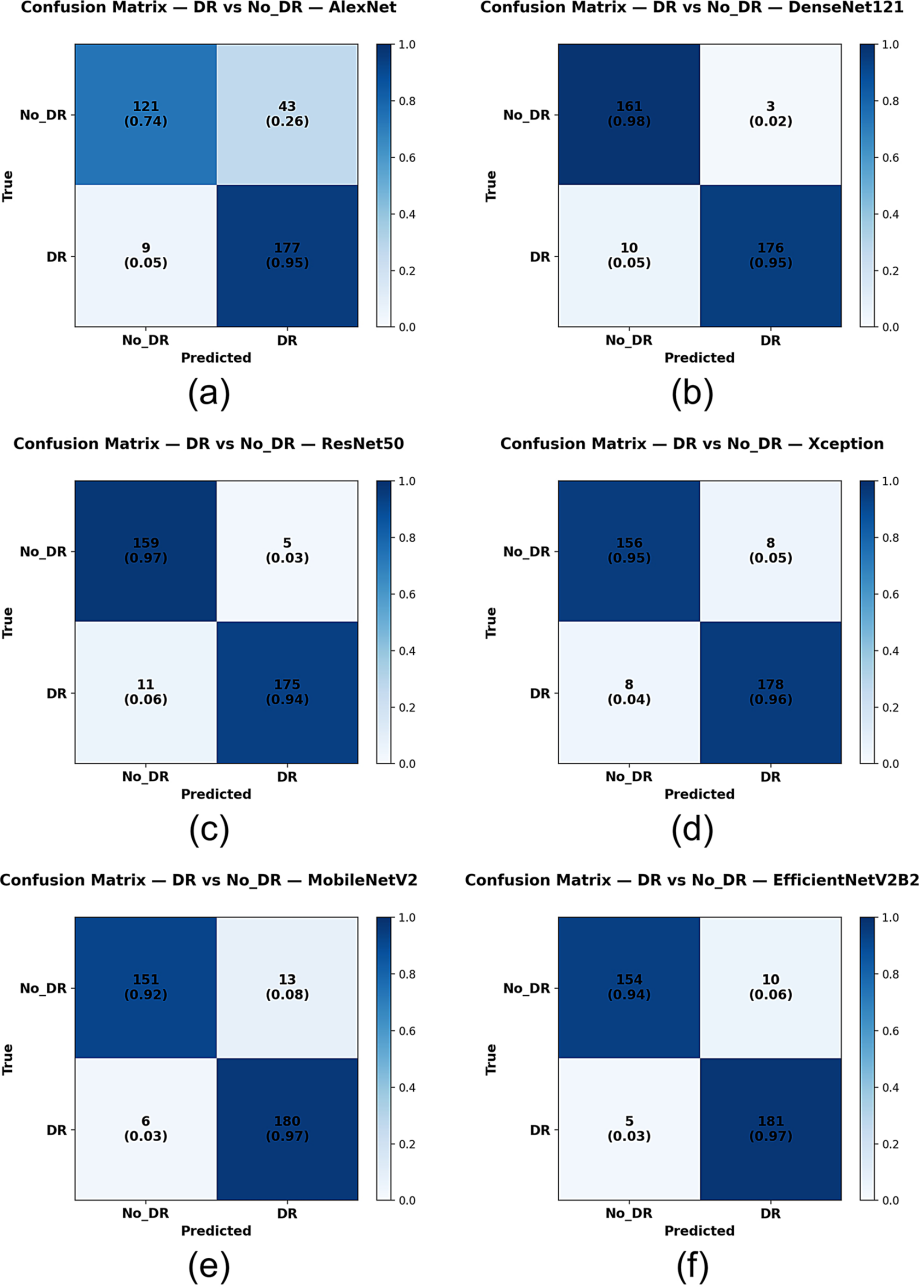

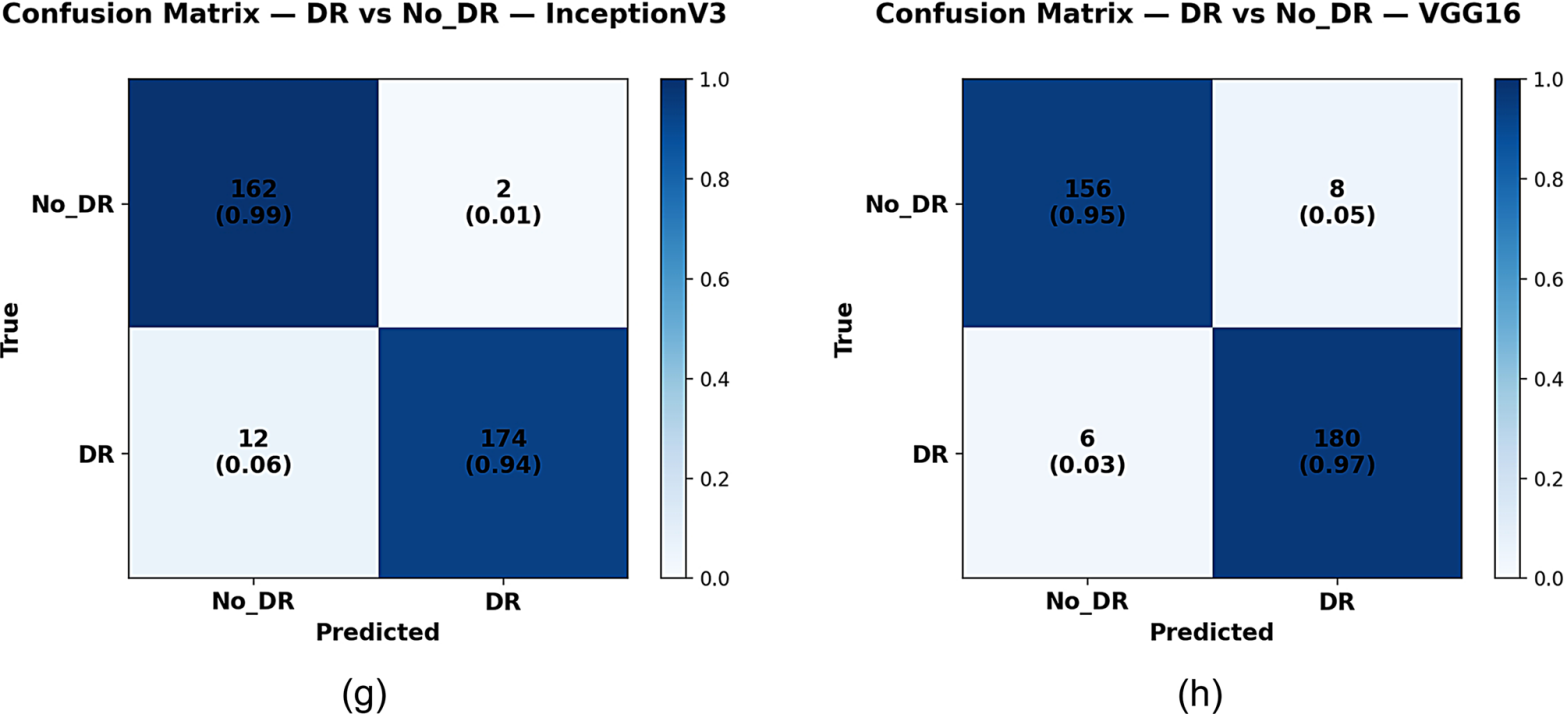
Confusion matrices (normalized) for binary DR detection (DR vs No DR) using DL architectures: (**a**) AlexNet; (**b**) DenseNet121; (**c**) ResNet50; (**d**) Xception; (**e**) MobileNetV2; (**f**) EfficientNetV2B2; (**g**) InceptionV3; (**h**) VGG16

**Fig. 12:**
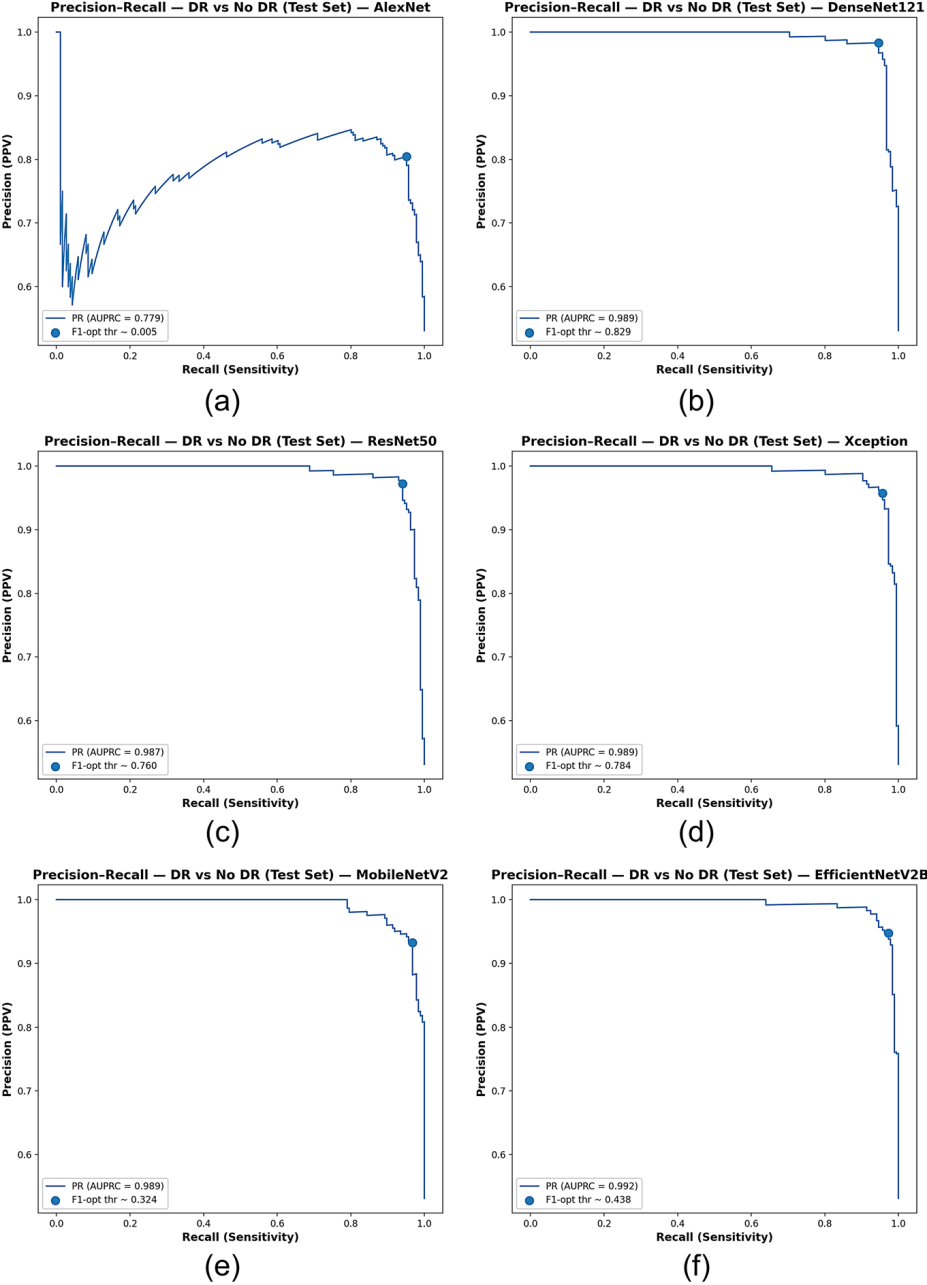

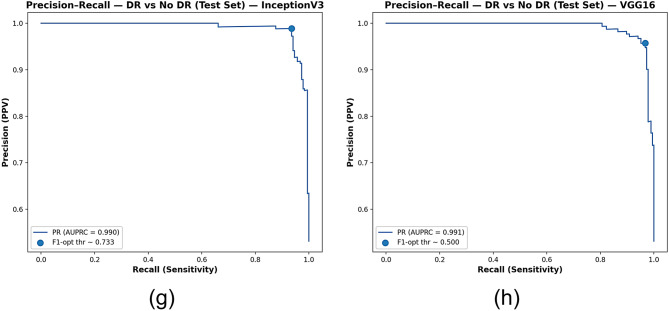
Precision–Recall curves on the test set for binary DR detection (DR vs No DR) using DL architectures: (**a**) AlexNet; (**b**) DenseNet121; (**c**) ResNet50; (**d**) Xception; (**e**) MobileNetV2; (**f**) EfficientNetV2B2; (**g**) InceptionV3; (**h**) VGG16

**Fig. 13:**
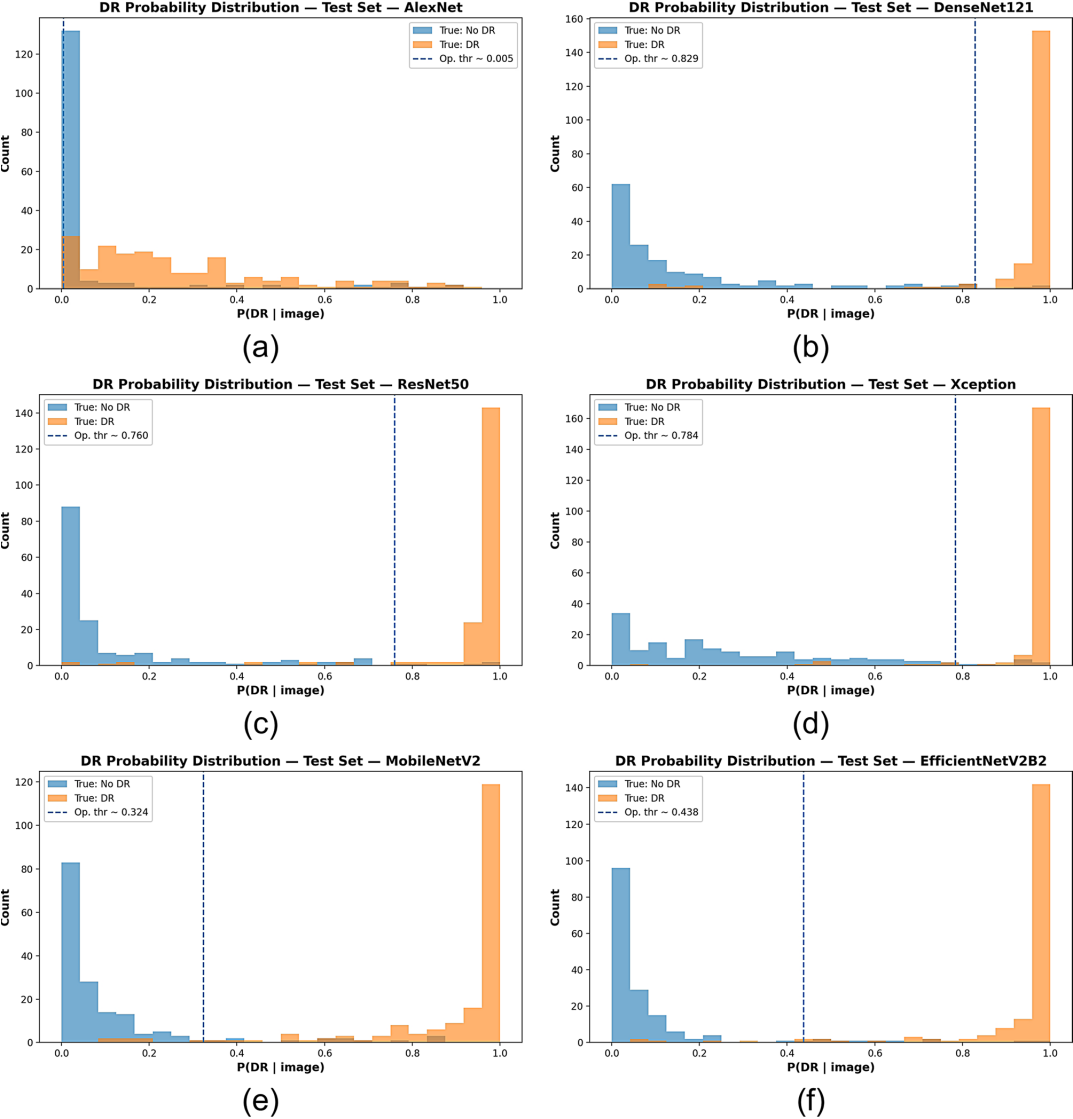

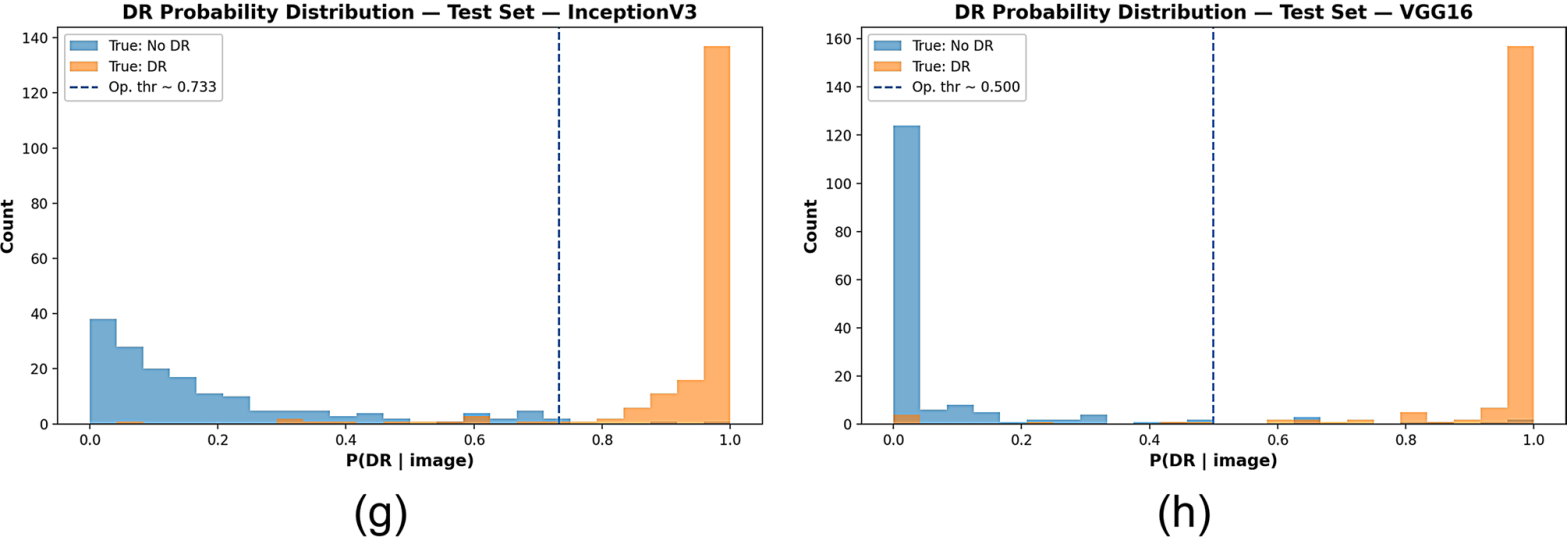
Probability distributions of *P (DR / image)* on the test set for binary detection (DR vs No DR) using DL architectures: (**a**) AlexNet; (**b**) DenseNet121; (**c**) ResNet50; (**d**) Xception; (**e**) MobileNetV2; (**f**) EfficientNetV2B2; (**g**) InceptionV3; (**h**) VGG16. Vertical dashed line indicates the operating threshold

**Fig. 14:**
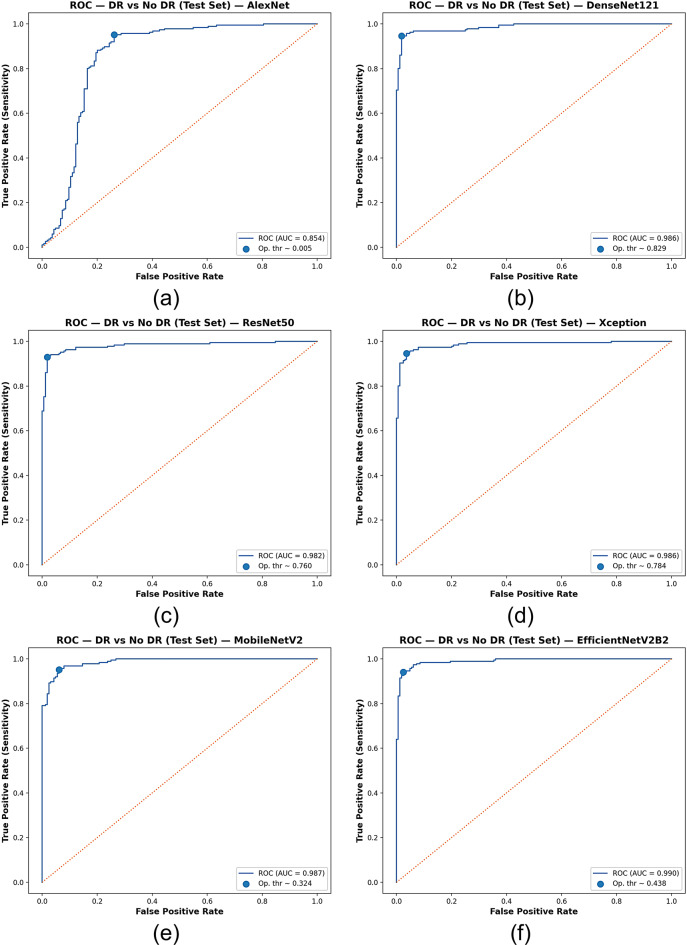

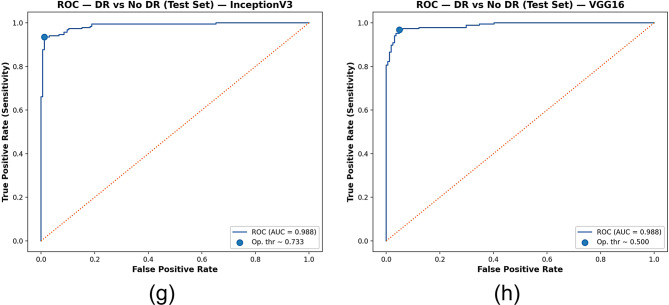
ROC curves on the test set for binary DR detection (DR vs No DR) using DL architectures: (**a**) AlexNet; (**b**) DenseNet121; (**c**) ResNet50; (**d**) Xception; (**e**) MobileNetV2; (**f**) EfficientNetV2B2; (**g**) InceptionV3; (**h**) VGG16

From the multiclass predictions, a binary endpoint was derived in which DR comprised “Mild NPDR, Moderate NPDR, Severe NPDR, PDR” and “No DR” was retained as a separate class. Decision thresholds were swept over $P\left( {DR\mid image} \right)$ on the test-set predictions as a post-hoc operating-point analysis, and the reported operating point was selected by maximizing the F1-score for the DR-positive class. Summary accuracy metrics are presented in Table 10, operating characteristics at the selected thresholds are reported in Table 11, and discrimination indices with the corresponding operating thresholds are provided in Table 12. The associated diagnostic visualizations—normalized confusion matrices, Precision–Recall curves, score distributions, and ROC curves—are shown in Figs. 11, 12, 13, and 14, respectively.

**Table 10:** Overall diagnostic performance (accuracy-based metrics) for binary DR detection

Algorithm	Accuracy	Balanced accuracy
AlexNet	0.8514	0.8447
DenseNet121	**0.9629**	**0.9640**
ResNet50	0.9543	0.9552
Xception	0.9543	0.9541
MobileNetV2	0.9457	0.9442
EfficientNetV2B2	0.9571	0.9561
InceptionV3	0.9600	0.9616
VGG16	0.9600	0.9595

**Table 11:** Operating characteristics at the selected threshold (sensitivity, specificity, predictive values, and F1-score)

Algorithm	Sensitivity (Recall, DR)	Specificity (No DR)	Precision (PPV, DR)	NPV (No DR)	F1-score (DR)
AlexNet	0.9516	0.7378	0.8045	0.9308	0.8719
DenseNet121	0.9462	0.9817	0.9832	0.9415	**0.9644**
ResNet50	0.9409	0.9695	0.9722	0.9353	0.9563
Xception	0.9570	0.9512	0.9570	0.9512	0.9570
MobileNetV2	0.9677	0.9207	0.9326	0.9618	0.9499
EfficientNetV2B2	**0.9731**	0.9390	0.9476	**0.9686**	0.9602
InceptionV3	0.9355	**0.9878**	**0.9886**	0.9310	0.9613
VGG16	0.9677	0.9512	0.9574	0.9630	0.9626

**Table 12:** Discrimination capacity and operating thresholds selected via F1-score maximization (DR as positive class)

Algorithm	Area under the ROC curve (AUROC)	AUPRC; DR positive	Operating threshold
AlexNet	0.8536	0.7788	0.0048
DenseNet121	0.9855	0.9889	**0.8285**
ResNet50	0.9817	0.9869	0.7600
Xception	0.9858	0.9892	0.7840
MobileNetV2	0.9869	0.9890	0.3235
EfficientNetV2B2	**0.9898**	**0.9915**	0.4378
InceptionV3	0.9876	0.9903	0.7328
VGG16	0.9881	0.9908	0.4996

All modern DL architectures across various frameworks attained an accuracy of ≥0.94 and a balanced accuracy of ≥0.94, as shown in Table 10. The most proficient models were DenseNet121 (0.9629/0.9640), EfficientNetV2B2 (0.9571/0.9561), InceptionV3 (0.9600/0.9616), VGG16 (0.9600/0.9595), and ResNet50 (0.9543/0.9552), while AlexNet underperformed (0.8514/0.8447). These differences correspond to architectural competence, extensibility, and representational power. Figure 11 shows the normalized confusion matrices, which enables to look into the corresponding error patterns.

As described in Table 11, separate operating profiles based on F1-optimal thresholds were also identified. MobileNetV2 (sensitivity 0.9677; specificity 0.9207) and EfficientNetV2B2 (0.9731; 0.9390) were also observed to be high-sensitivity screeners making them more suitable for screening/triage settings where missed disease should be minimized. InceptionV3 (specificity 0.9878; PPV 0.9886; sensitivity 0.9355) represented high-specificity confirmers, in line with the confirmatory/second-read role where false positives are costly downstream. This study achieved balanced operating points with DenseNet121 (0.9462/0.9817; F1 0.9644), ResNet50 (0.9409/0.9695; F1 0.9563), Xception (0.9570/0.9512; F1 0.9570), and VGG16 (0.9677/0.9512; F1 0.9626). On the other hand, AlexNet demonstrated asymmetric behavior (sensitivity 0.9516; specificity 0.7378) resulted in lower PPV and Negative Predictive Value (NPV), decreasing the overall accuracy and F1. These behaviors are visually confirmed by the confusion patterns as shown in Fig. 11.

Discrimination capacity was uniformly high among modern backbones, with **AUROC ≈ 0.982–0.990** and Area Under the Precision–Recall Curve (AUPRC) ≈ 0.987–0.992, as reported in Table 12. *EfficientNetV2B2* achieved the highest AUROC (0.9898) and AUPRC (0.9915), closely followed by *InceptionV3*, *VGG16*, *MobileNetV2*, *Xception*, and *DenseNet121*, while *AlexNet* trailed (AUROC 0.8536; AUPRC 0.7788). The PR curves cluster near the upper-right region for the stronger models—indicating high PPV at high recall—as shown in Fig. 12, and the ROC curves illustrate a similar ranking, as shown in Fig. 14.

The P (DR|image) distributions indicate obvious score differences between the modern architectures between DR and No DR with insignificant overlap and sharply bimodal behavior of EfficientNetV2B2 and InceptionV3 in Fig. 13. It is also worth noting that the selected operating thresholds differ significantly between DenseNet121 and MobileNetV2 with a difference of about 0.83 and 0.32 respectively. The very low threshold of the AlexNet (approximately 0.005) shows that it is not well-calibrated, and its sensitivity is high at the expense of the number of false positives; it can be observed in the PR behavior as well as the probability histograms, which are presented in Figs. 12 and 13.

Combined, the findings suggest that either of DenseNet121, EfficientNetV2B2, InceptionV3, VGG16, ResNet50, or Xception can be used to perform the screening of DR/No DR, with all of them achieving the accuracy, F1, AUROC, and AUPRC above 0.95 as shown in Tables 10, 11 and 12. Besides, threshold-based workflow tuning is feasible: sensitivity can be focused on first-line screening for example (MobileNetV2 or EfficientNetV2B2), whereas specificity can be focused on referral confirmation for example (InceptionV3), as it can be seen in Table 11. Finally, because optimal thresholds differ markedly across architectures, calibration matters; deployment should include post-training calibration and site-specific threshold selection informed by validation data, as supported by the score distributions and operating points shown in Figs. 13 and 14.

The reported operating thresholds correspond to the F1-optimal points obtained by threshold sweeping over the predicted probabilities, and therefore reflect a balance between sensitivity and precision rather than purely sensitivity–specificity trade-offs.

### Clinically-oriented binary endpoints

To further enhance clinical relevance, two additional binary endpoints were evaluated based on clinically established referral criteria. Referable Diabetic Retinopathy (RDR) was defined as Moderate NPDR or worse (Moderate NPDR, Severe NPDR, and PDR), corresponding to cases requiring referral to an ophthalmologist. Vision-Threatening Diabetic Retinopathy (VTDR) was defined as Severe NPDR or worse (Severe NPDR and PDR), corresponding to cases requiring urgent specialist intervention.

For both tasks, predictions from the five-class models were aggregated into binary probabilities, and decision thresholds were determined by maximizing the F1-score on the test set, consistent with the primary binary analysis.

As shown in Tables 13 and 14, all modern architectures maintained high discrimination performance across both clinically-oriented tasks, with consistently strong AUROC and AUPRC values. Performance trends were consistent with the DR vs No DR setting, with deeper architectures demonstrating robust generalization and stable operating characteristics. These results confirm that the proposed framework can be readily adapted to clinically actionable screening and referral scenarios without retraining.

**Table 13:** Discrimination capacity and F1-optimized operating thresholds for RDR detection

Algorithm	Area under the ROC curve (AUROC)	AUPRC; RDR positive	Operating threshold
AlexNet	0.7872	0.6272	0.0048
DenseNet121	0.9760	0.9700	0.2641
ResNet50	0.9733	0.9573	0.3385
Xception	0.9640	0.9526	0.4940
MobileNetV2	0.9682	0.9577	0.4793
EfficientNetV2B2	0.9827	0.9767	0.3646
InceptionV3	0.9745	0.9664	0.3917
VGG16	0.9759	0.9628	0.2189

**Table 14:** Discrimination capacity and F1-optimized operating thresholds for VTDR detection

Algorithm	Area under the ROC curve (AUROC)	AUPRC; VTDR positive	Operating threshold
AlexNet	0.7085	0.2185	0.0019
DenseNet121	0.9083	0.6021	0.5361
ResNet50	0.8952	0.5426	0.3550
Xception	0.9146	0.6291	0.4678
MobileNetV2	0.9119	0.6584	0.1278
EfficientNetV2B2	0.9146	0.6130	0.4459
InceptionV3	0.8974	0.5840	0.2281
VGG16	0.9039	0.5671	0.2490

These endpoints were derived from the same trained models without additional retraining by aggregating class probabilities.

**Fig. 15:**
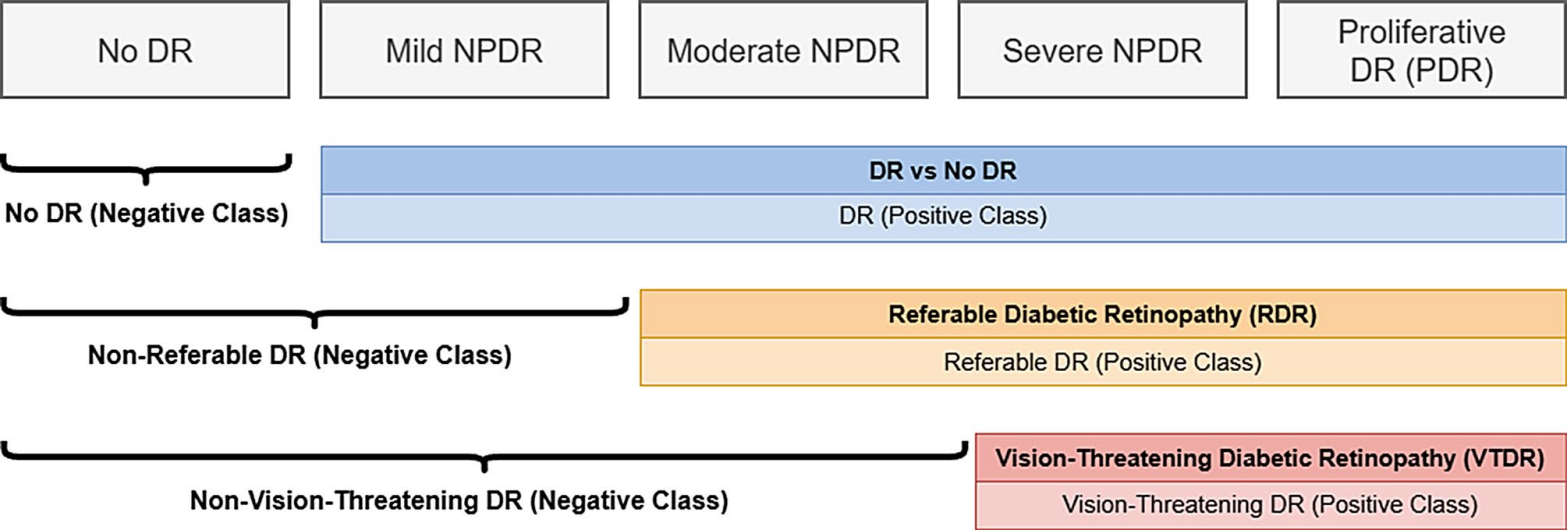
Definition of clinically relevant binary endpoints for DR screening, illustrating DR vs No DR, RDR, and VTDR across the five-stage disease severity scale

Figure 15 illustrates the formulation of clinically oriented binary endpoints derived from the five-level DR grading scale. The standard DR vs No DR task groups all disease stages (Mild NPDR and above) as the positive class. In contrast, RDR considers Moderate NPDR and more advanced stages as positive, aligning with referral decisions, while VTDR restricts the positive class to Severe NPDR and PDR, corresponding to cases requiring urgent intervention. These definitions reflect progressively stricter clinical thresholds and support task-specific screening and triage strategies.

### Comparison across models

**Fig. 16:**
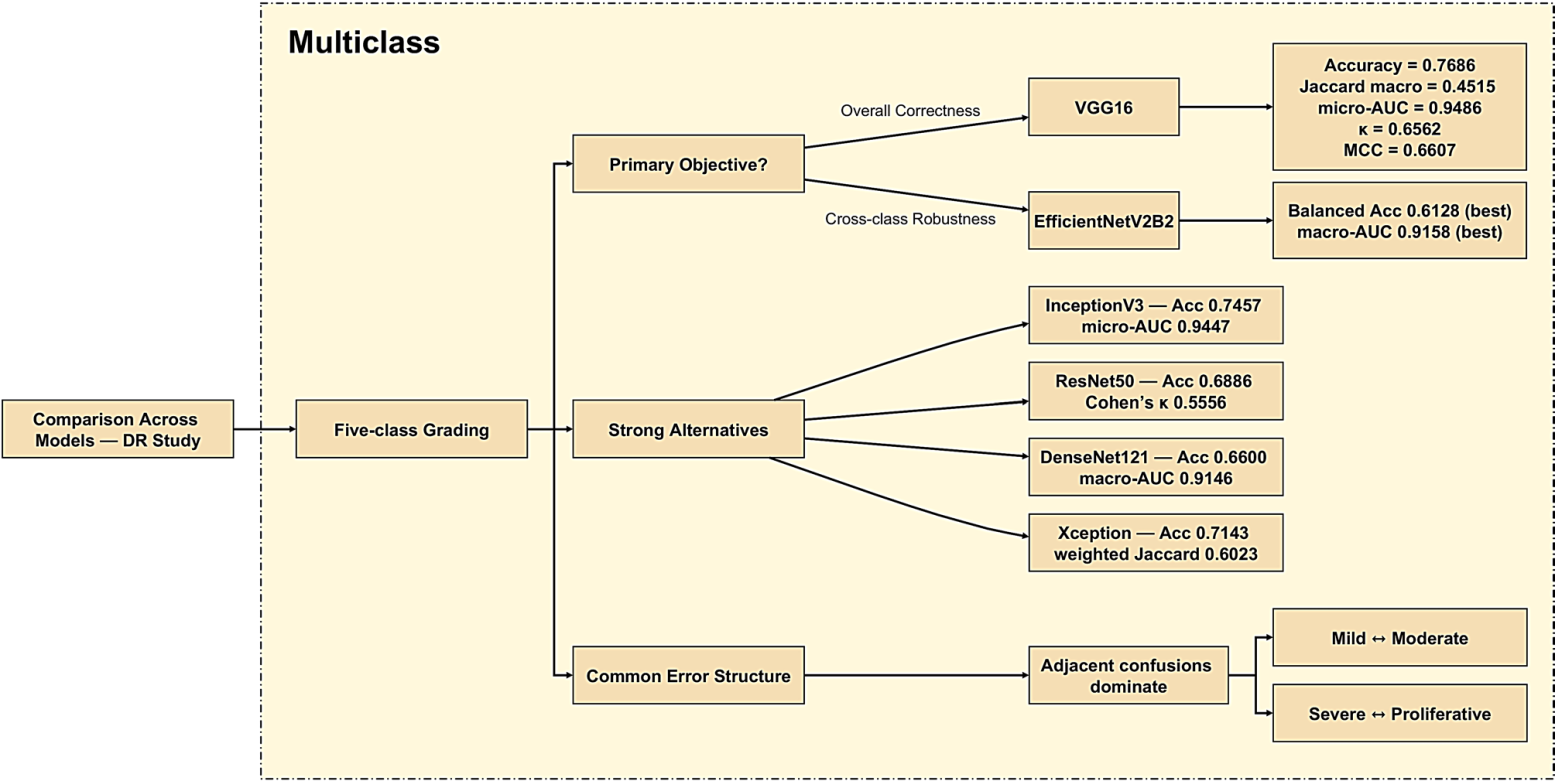
Comparative performance of DL architectures in multiclass DR grading, highlighting overall accuracy, balanced accuracy, and common error structures across severity levels

**Fig. 17:**
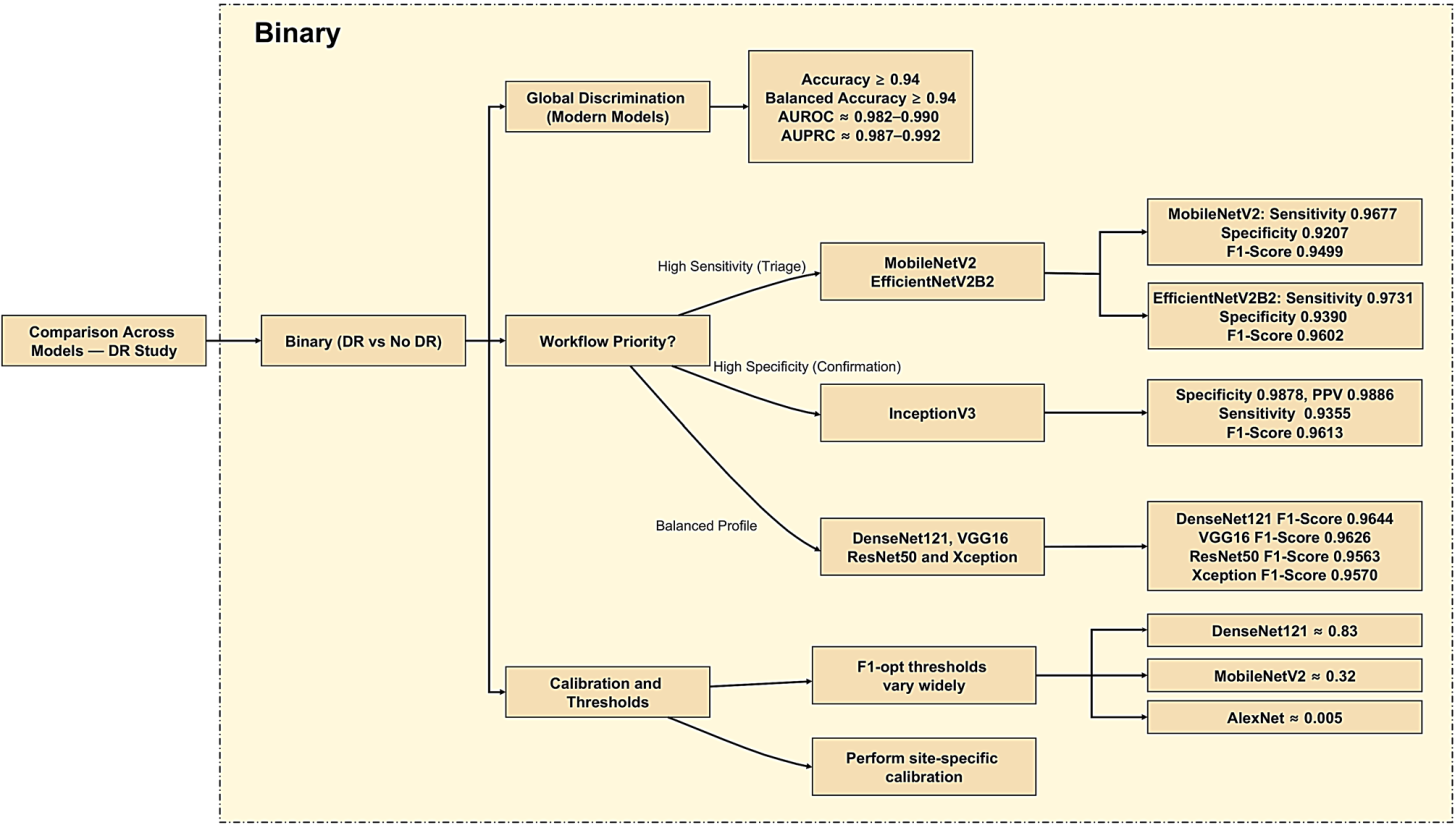
Binary classification results (DR vs No DR) across architectures, illustrating global discrimination capacity, workflow-oriented operating points, and calibration differences

**Fig. 18:**
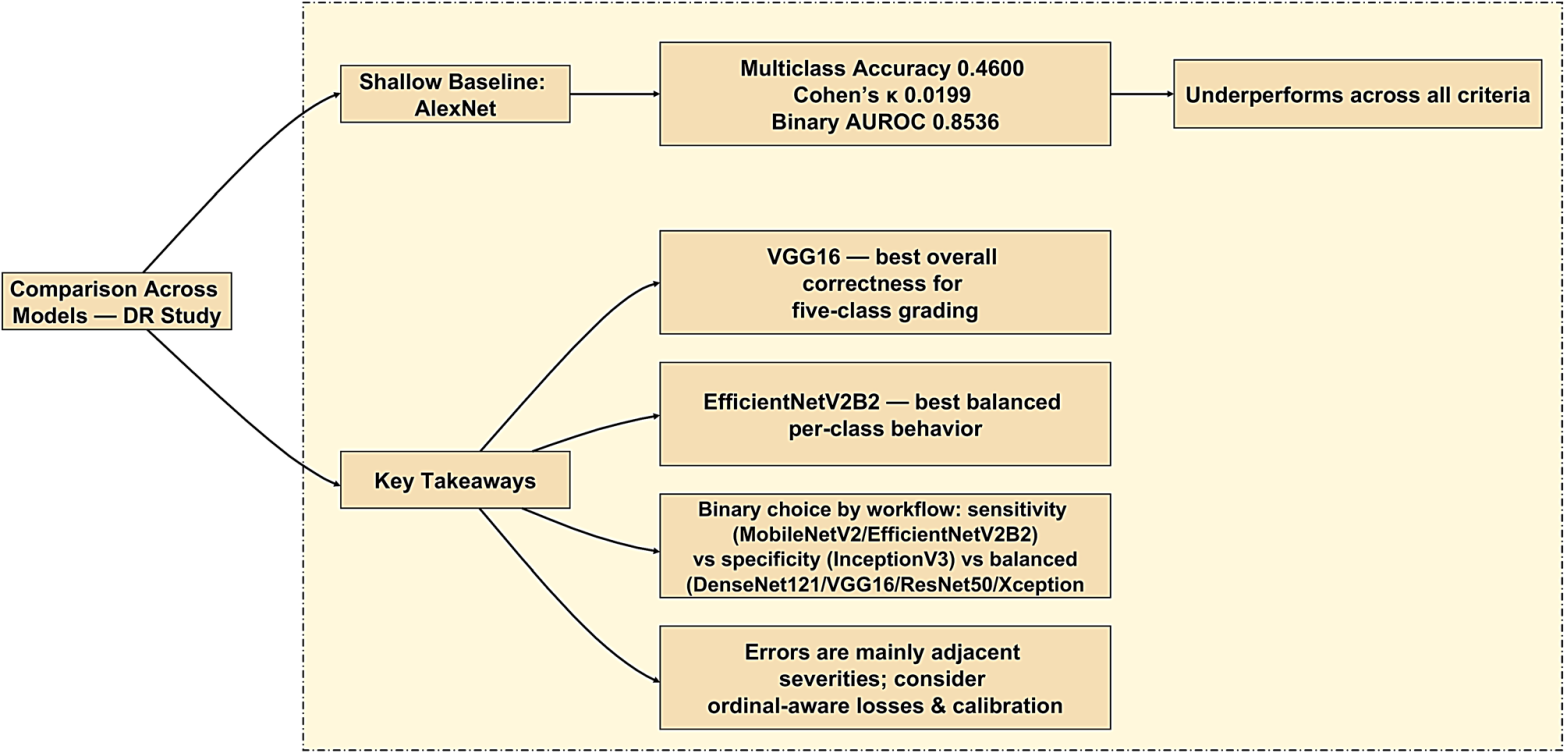
Baseline versus advanced architectures in DR detection, summarizing AlexNet’s underperformance and key takeaways from modern DL backbones

VGG16 model gives the highest overall correctness of the five-class grading, whereas EfficientNetV2B2 model has the most balanced per-class performance; InceptionV3, ResNet50, DenseNet121, and Xception are all viable options with errors distributed focused mainly between nearby severities, as Fig. 16 shows. Throughout the binary environment, workflow-driven decisions are supported by all modern backbones with uniform high discrimination: MobileNetV2 and EfficientNetV2B2 are sensitive to triage, InceptionV3 to specificity to confirmation, and DenseNet121, VGG16, ResNet50, and Xception to balanced profiles; the best thresholds across models are also significantly different, as demonstrated in Fig. 17. On the other hand, the AlexNet shallow baseline has a disadvantage in criteria, which underlines the merits of the existing architectures and careful calibration, as summarized in Fig. 18.

## Future work and dataset expansion

This study used a single public fundus-image dataset to compare deep learning models. However, relying on just one dataset may limit the model’s performance across different populations, cameras, and acquisition settings. Future work should expand the proposed pipeline to include more public DR datasets from various sources, annotations, and imaging types. As shown in Table 15, datasets like DDR, IDRiD, MFIDDR, and MMRDR each offer unique advantages. DDR improves performance across institutions. IDRiD aids in lesion-aware and explainable modeling. MFIDDR supports multi-view learning. MMRDR facilitates multimodal learning with CFP, OCT, and UWF imaging. Furthermore, future research could investigate new deep learning methods such as transformer-based models, hybrid CNN and ViT architectures, and ensemble approaches to boost performance. It is also essential to explore external validation across datasets, domain adaptation, ordinal learning strategies, and post-training calibration to improve reliability and practical use.

**Table 15:** Public DR datasets for future work

Dataset	Reference	Year	Size & Modality	Key Characteristics	Relevance to This Study	Future Integration Value
DDR	[34]	2019	13,673 fundus images	Clinical dataset; 6-class DR grading; includes lesion annotations (subset)	Not used in this study	Improves robustness across populations and imaging conditions
IDRiD	[35]	2018	Open access; color fundus photography	Representative of an Indian population; includes pixel-level lesion annotations and DR/DME grading labels (ISBI 2018).	Not used in this study	Supports lesion-aware modeling, explainability, and improved generalization.
MFIDDR	[36]	2026	34,452 fundus images; multi-view (4-view)	First large-scale 4-view dataset; expert-labeled; includes clinical info	Not used in this study	Improves generalization via multi-view learning
MMRDR	[37]	2026	Large-scale; multimodal (CFP, OCT, UWF)	Multimodal dataset with lesion-level annotations and DR/DME grading	Not used in this study	Enhances generalization and supports multimodal learning

## Conclusion

This study developed a complete and reproducible framework for DR screening and grading and presents a head-to-head comparison of eight popular CNN architectures trained and evaluated on the same conditions. The performance of modern backbones was always higher than a shallow baseline, and their error distributions were in line with clinical expectations- most misclassifications were between adjacent levels of severity. VGG16 was the most successful in the overall correctness of five-class grading, whereas EfficientNetV2B2 was better at class balance and macro-level discrimination. Reformulated as a binary choice, all new architectures were found to have high discrimination and high operating characteristics, providing deployment options that are either sensitivity-focused (as in the case of first-line triage) or specificity-focused (as in the case of confirmatory reads). Besides crude accuracy, three design alternatives were identified as relatively effective across models: robust augmentations, uniform sampling, and incremental fine-tuning of upper layers. Post-hoc threshold analysis also revealed that there is a significant difference between optimal operating points according to the backbone, highlighting the significance of local calibration before clinical usage. Based on these results, the future directions of the research will involve multi-site validation, domain adaptation between cameras and populations, explicit ordinal or cost sensitive objectives that are aware of DR progression, robust calibration with continuous drift monitoring, model explainability to support clinician acceptance, plus integration with teleophthalmology processes in resource constrained systems. These measures can ensure that automated systems can increase screening capacity significantly, decrease time-to-referral, and contribute to preventing unnecessary vision loss.

## Data Availability

The datasets used and/or analysed during the current study available from the corresponding author on reasonable request.

## Abbreviations

AI: Artificial Intelligence
AIDRSS: AI-based DR Screening System
APTOS: Asia Pacific Tele-Ophthalmology Society
AUC: Area Under the Curve
AUROC: Area Under the Receiver Operating Characteristic Curve
AUPRC: Area Under the Precision–Recall Curve
CC0: Creative Commons Zero
CNN: Convolutional Neural Network
DenseNet121: Densely Connected Convolutional Network 121
DL: Deep Learning
DR: Diabetic Retinopathy
EfficientNetV2B2: Efficient Network Version 2 B2
ETDRS: Early Treatment Diabetic Retinopathy Study
FPR: False Positive Rate
ICDR: International Clinical Diabetic Retinopathy
InceptionV3: Inception Version 3
LBP: Local Binary Patterns
LMICs: Low- and Middle-Income Countries
MCC: Matthews Correlation Coefficient
MobileNetV2: Mobile Network Version 2
NPDR: Non-Proliferative Diabetic Retinopathy
NPV: Negative Predictive Value
PDR: Proliferative Diabetic Retinopathy
PPV: Positive Predictive Value
QWK: Quadratic-Weighted Kappa
RDR: Referable Diabetic Retinopathy
ResNet50: Residual Network 50
ROC: Receiver Operating Characteristic
TPR: True Positive Rate
VEGF: Vascular Endothelial Growth Factor
VGG16: Visual Geometry Group 16
VTDR: Vision-Threatening Diabetic Retinopathy
Xception: eXtreme Inception
